# Investigation into the efficiency of diverse *N*‐linking oligosaccharyltransferases for glycoengineering using a standardised cell‐free assay

**DOI:** 10.1111/1751-7915.14480

**Published:** 2024-06-10

**Authors:** Burhan Lehri, Elizabeth Atkins, Timothy A. Scott, Sherif Abouelhadid, Brendan W. Wren, Jon Cuccui

**Affiliations:** ^1^ Department of Infection Biology London School of Hygiene and Tropical Medicine London UK; ^2^ Department of Medicine, Cambridge Institute of Therapeutic Immunology & Infectious Disease (CITIID), Jeffrey Cheah Biomedical Centre University of Cambridge Cambridge UK

## Abstract

The application of bacterial oligosaccharyltransferases (OSTs) such as the *Campylobacter jejuni* PglB for glycoengineering has attracted considerable interest in glycoengineering and glycoconjugate vaccine development. However, PglB has limited specificity for glycans that can be transferred to candidate proteins, which along with other factors is dependent on the reducing end sugar of glycans. In this study, we developed a cell‐free glycosylation assay that offers the speed and simplicity of a ‘yes’ or ‘no’ determination. Using the assay, we tested the activity of eleven PglBs from *Campylobacter* species and more distantly related bacteria. The following assorted glycans with diverse reducing end sugars were tested for transfer, including *Streptococcus pneumoniae* capsule serotype 4, *Salmonella* enterica serovar Typhimurium O antigen (B1), *Francisella tularensis* O antigen, *Escherichia coli* O9 antigen and *Campylobacter jejuni* heptasaccharide. Interestingly, while PglBs from the same genus showed high activity, whereas divergent PglBs differed in their transfer of glycans to an acceptor protein. Notably for glycoengineering purposes, *Campylobacter hepaticus* and *Campylobacter subantarcticus* PglBs showed high glycosylation efficiency, with *C. hepaticus* PglB potentially being useful for glycoconjugate vaccine production. This study demonstrates the versatility of the cell‐free assay in rapidly assessing an OST to couple glycan/carrier protein combinations and lays the foundation for future screening of PglBs by linking amino acid similarity to glycosyltransferase activity.

## Graphical abstract

This article introduces a simplified version of a cell‐free glycosylation assay, known as Three‐in‐One Pot (TiOP), designed for the rapid screening of bacterial oligosaccharyltransferases (OSTs). Our assay evaluated multiple OSTs, in conjunction with a diverse array of glycans, including some expressed natively. Among our key findings were the remarkable glycosylation efficiencies of OSTs isolated from *Campylobacter hepaticus* and *Campylobacter subantarcticus*. We also established a correlation between amino acid sequence similarity of bacterial OSTs and their ability to glycosylate. Additionally, the assays capability to test glycans without the need for recombinant expression could be a notable advancement. Our findings provide advancements for bacterial glycoconjugate vaccine development, as it provides a platform for screening OSTs and their glycosylation in a rapid manner.

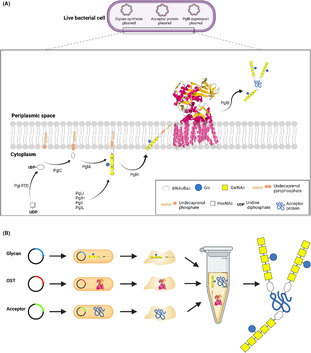

## INTRODUCTION

**FIGURE 1:**
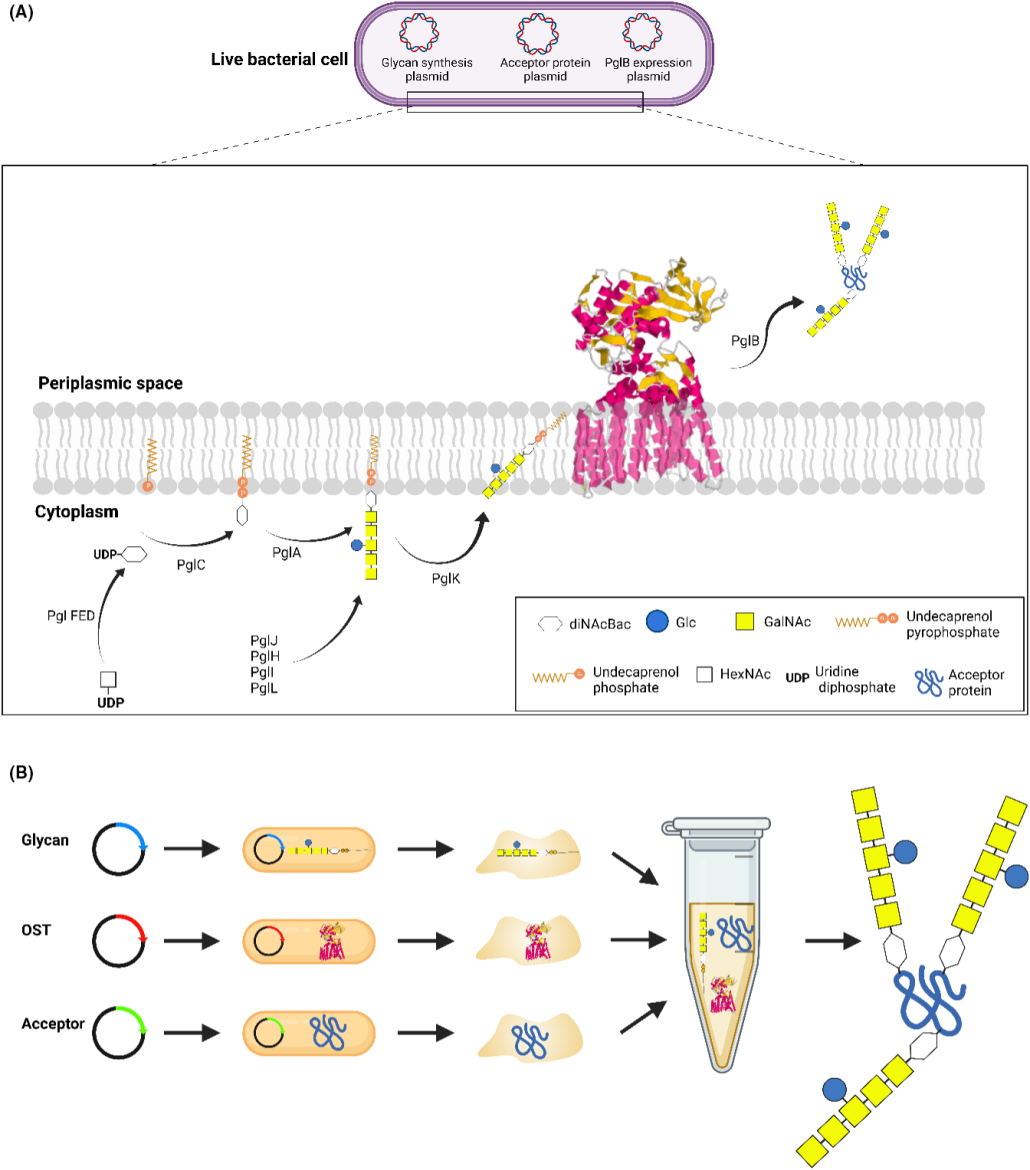
Overview of the bacterial glycosylation machinery. (A) PGCT, a living cell where several genes involved in glycan, OST and protein synthesis are induced to generate a proteoglycan within the periplasmic space. (B) TiOP overview where substrates for glycosylation are expressed independently and then combined to generate a glycopeptide.

Using bacteria to couple an oligosaccharide to an immunogenic protein heralds a cost‐effective approach for glycoconjugate vaccine production, offering a potential alternative to more expensive chemical techniques. During the structural and functional characterisation of the *Campylobacter jejuni N*‐linked glycosylation system, the *C. jejuni* protein glycosylation locus (*pgl*) was transferred to *Escherichia coli* and was the first demonstration of the expression of recombinant glycoproteins in a bacterial cell; paving the way for glycoconjugate vaccine development (Linton et al., 2005). The *C. jejuni pgl* locus encodes glycosyltransferases that build a heptasaccharide structure on the lipid carrier undecaprenol pyrophosphate, a flippase then transfers the heptasaccharide from the cytoplasm to the periplasm, and an oligosacharyltransferase (OST), PglB, transfers the assembled glycan from the lipid intermediate onto an asparagine residue within the sequon D/E‐X‐N‐X‐S/T, where X can be any amino acid residue other than proline (Wacker et al., 2006) (Figure 1A). The *C. jejuni* PglB, in comparison to other OSTs, has a relatively relaxed specificity for transferring glycans with different reducing end sugars other than that of its native diacetylbacillosamine (diNAcBac) (Wacker et al., 2006). The discovery of *C. jejuni* PglB signalled a new era in glycoengineering with the potential to clone and express diverse glycostructure combinations in *E. coli* cells (Langdon et al., 2009). The main application of this bacterial glycoengineering approach is to develop a rapid, reliable, and cost‐effective means of glycoconjugate vaccine production, with vaccines currently undergoing phase I and II clinical trials (Dow et al., 2020; Huttner et al., 2017; LimmaTech Biologics AG, 2018, 2022). This approach, where different proteins/peptides can be coupled to glycans within a bacterial cell to produce tailor‐made glycostructures, has been termed Protein Glycan Coupling Technology (PGCT) or bioconjugation (Dow et al., 2020). The most studied PGCT bacterial OST to date is the original *C. jejuni* PglB but with the advent of whole genome sequencing, PglB homologues have been identified in other species, particularly in the *Campylobacter* genus, some of which have been characterised. To date, other PglBs as promiscuous or as efficient in glycoconjugation as the original *C. jejuni* PglB have not been identified (Faridmoayer et al., 2008; Kay et al., 2019; Knoot et al., 2021). Such novel PglBs would be invaluable for glycoengineering.

Protein glycosylation is a complex process, where the components of the biological reaction require a balance between the expression of glycan, protein and OST (Figure 1A), needing the development of screening assays to test the aforementioned variables. In this study, the cell‐free glycoprotein synthesis (CFGpS) technology (Jaroentomeechai et al., 2018) was simplified to remove the transcription–translation step with its complex reaction buffer consisting of 36 amino acids, rNTPs and cofactors to only the manganese and membrane‐mimetic detergent required for efficient OST function. This simplified CFGpS, referred to as Three‐in‐One Pot (TiOP), was used to screen several bacterial OSTs and glycans derived from bacterial species (Figure 1B). TiOP allows independent expression of each component (acceptor protein, glycan and OST) required for glycosylation, removing the complexity of expressing them concurrently in live bacterial cells (Figure 1A). In addition, TiOP allows the use of natively expressed glycan, avoiding the need for cloning and recombinantly expressing large glycan loci into *E. coli*, useful for rapid screening prior to the use of PGCT for vaccine development.

**FIGURE 2:**
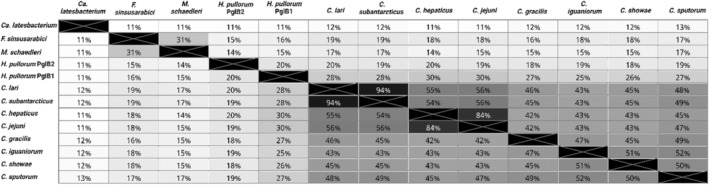
PglB amino acid sequence similarity heat map. Showing amino acid percent similarity alignment using Clustal Omega, the darker the colour of the map the higher the amino acid percent similarity between OSTs.

For this study, phylogenetically diverse PglBs were selected for testing their ability to transfer an assortment of glycans including O antigens, capsular polysaccharides and glycosylation systems (Figure 2). *C. subantarcticus* was chosen due to high sequence similarity to *C. lari* PglB (94%, Figure 2), which has a known X‐ray crystallography structure (Lizak et al., 2011; Napiórkowska et al., 2017). *Campylobacter showae*, *C. sputorum* (Etoh et al., 1993), *Campylobacter iguaniorum* (Gilbert et al., 2015) and *Campylobacter gracilis* (Vandamme et al., 1995) PglBs all have amino acid sequence similarity to *C. jejuni* PglB ranging from 42 to 47% (Figure 2). *Campylobacter hepaticus* (Van et al., 2016) has high amino acid sequence similarity to *C. jejuni* PglB (84%, Figure 2). *Helicobacter pullorum* PglB1 and PglB2 have 20% amino acid sequence similarity to each other, and 30% and 19% sequence similarity to *C. jejuni* PglB, respectively. The divergent OSTs from *Mucispirillum schaedleri*, *Flexistipes sinusarabici*, and *Candidatus latescibacterium* were chosen due to their low amino acid sequence similarity to *C. jejuni* PglB (11–18%) (Figure 2). PglB from Ca. *latescibacterium* was the most genetically distant from *C. jejuni* PglB in terms of sequence similarity and has a higher amino acid sequence similarity to the eukaryotic STT3A and STT3B binding subunit than *C. jejuni* PglB, 56.1% and 54.4%, respectively, as opposed to 29.1–37.1% similarity to other PglBs tested including *C. jejuni*.

To assess the specificity of the selected OSTs, a diverse range of glycans were tested, as shown in Table 1. *Salmonella enterica serovar* Typhimurium (*S*. Typhimurium) and *E. coli* O9 glycans, were obtained from native bacteria, while others were expressed recombinantly from different *E. coli* glycoengineering host strains.

**TABLE 1:** Summary of the glycans tested.

Glycan	Reducing end sugar	Original bacterial host	Expression host	Expression host type
*Campylobacter jejuni* heptasaccharide	GlcNAc and DiNAcBac	*C. jejuni*	*E. coli* CLM24	*wecA* gene positive, *waal* gene knockout
*Campylobacter jejuni* heptasaccharide	DiNAcBac	*C. jejuni*	*E. coli* SDBI	*wecA*, *waal* gene knockout
*Streptococcus pneumoniae* serotype 4	GlcNAc and GalNAc	*S. pneumoniae*	*E. coli* W311O	
*Salmonella enterica* serovar Typhimurium O antigen (B1)	Gal	*S*. Typhimurium SL3749	*S*. Typhimurium SL3749	*waaL* gene knockout
*Escherichia coli* O9	GlcNAc	*E. coli* O9	*E. coli* E69	
*Francisella tularensis*	GlcNAc and Qui4NFm	*F. tularensis*	*E. coli* DH5alpha	

Here we test different OSTs to identify potential glycosylation transfer patterns based on amino acid percent similarity, and to identify more promiscuous OST(s) that may glycosylate proteins more efficiently than *C. jejuni* PglB. The study also aims to demonstrate the utility of a simplified CFGpS (TiOP) in rapidly selecting substrates for bacterial glycoengineering and comparing its potential as a screening tool alongside PGCT.

## EXPERIMENTAL PROCEDURES

### OST constructs

Oligosaccharyltransferases were initially transformed into 10‐beta electrocompetent *E. coli* cells (NEB). For OST expression, *E. coli* strain SDB1 (*wecA* negative to determine glycosylation without GlcNAc (Garcia‐Quintanilla et al., 2014; Linton et al., 2005) was transformed with vector pEXT21, along with other *E. coli* strains, where specified, including CLM24 and DH5α. PglB transformation was conducted using electroporation following manufacturer guidelines with a Gene Pulser Xcell electroporation system (Bio‐Rad) at 2.5 kV in 0.2 cm electroporation cuvettes (Bio‐Rad). Transformation was confirmed by colony PCR using primers shown in Table S1. PCR conditions were: 95°C initial denaturation, 30 cycles of 95°C for 15 s, 65°C for 15, 30 and 40 s, respectively, followed by 72°C for 5 min. Sanger sequencing was undertaken to validate the correct OST sequences.

### Cell culture and protein/OST/glycan expression

For the cell‐free assay, *E. coli* strains were plated on Luria‐Bertani agar (Fisher Scientific) and grown overnight at 37°C. For OST expression, strains were grown in 10 mL 2YPTG broth (10 g/L Yeast extract, 16 g/L Tryptone, 5 g/L NaCl, 7 g/L K_2_HPO_2_, 3 g/L KH_2_PO_4_, 18 g/L glucose; pH 7.2) overnight at 37°C. This was then used to inoculate 2 L of 2YPTG broth and grown at 30°C until an OD_600_ 0.5–0.6 nm was reached. Samples were then induced with 1 mM Isopropyl β‐D‐1‐thiogalactopyranoside (IPTG) (Fisher reagents). Acceptor protein EPA (10 sequons) with a hexa histidine tag was expressed from plasmid pEC415 (Marshall et al., 2018) in *E. coli* SDB1 under conditions similar to OSTs, although in Luria‐Bertani (Miller) broth (Fisher reagents) with 0.2% L‐arabinose inducer (Fisher reagents) and growth was 30°C overnight and 110 rpm. AcrA with two glycosylation sequons was expressed in *E. coli* SDB1 from plasmid pIPE30 using the same conditions as EPA except, induction was with 1 mM IPTG. Glycan donors were grown in the appropriate media for each cell line and/or construct: 2YTPG for pACYC*pglB::kan* (Linton et al., 2005) in CLM24 and SDB1, 2YT (16 g/L Tryptone, 10 g/L Yeast extract, 5 g/L NaCl, pH 7) for *S*. Typhimurium SL3749 (Roantree et al., 1977), and LB broth for all other constructs and cell lines.

Protein Glycan Coupling Technology was conducted in *E. coli* SDB1 with plasmid vectors pEXT21 (encoding *C. hepaticus* PglB, or *C. jejuni* PglB), pEC415 (encoding EPA with 10 acceptor sequons and hexa histidine tag), and pEXT22 (encoding *C. jejuni* heptasaccharide expression loci). *E. coli* SDB1 was grown at 37°C overnight on LB agar. Single colonies were selected to either grow overnight at 37°C and 120 rpm in LB broth or 30°C and 110 rpm in 2YPTG media. 0.5 mL of overnight culture was used to inoculate 9.5 mL of either LB broth or 2YPTG and incubated at 37°C, 120 rpm or 30°C and 110 rpm, respectively. Cultures were induced at OD_600_ 0.5–0.6 with 0.2% L‐arabinose and 1 mM IPTG, followed by additional 0.2% L‐arabinose induction after 5 h, then incubation continued overnight. The samples were then spun down and resuspended in NiNTA lysis buffer (50 mM NaH2PO4, 300 mM NaCl, 10 mM Imidazole, pH 8.0). All samples were OD_600_ matched to 1.0 using NiNTA lysis buffer.

### Lysis and protein pull‐down assays

For cell‐free assays: Cells were washed three times in S30 buffer (10 mM tris acetate, 14 mM magnesium acetate, 60 mM potassium acetate; pH 8.2). The samples were adjusted to 1 g/mL based on cell wet weight in S30 buffer and lysed with a pressure cell homogeniser (Stansted). Acceptor proteins were spun down at 15,000×*g* (Beckman Coulter) for 1 h at 4°C to remove cell debris and stored at −20°C prior to use. OST and glycan donors were aliquoted immediately after lysis and stored at −80°C.

For PGCT, samples were washed three times in NiNTA lysis buffer and made to an OD_600_ of 2.0 using a spectrophotometer. The samples were lysed using Lysing Matrix B beads (MP Biomedicals) in His wash buffer (50 mM NaH_2_PO_4_, 300 mM NaCl, 20 mM Imidazole, pH 8.0) using MP Biomedicals Fast Prep‐24 homogeniser (5 × 30 s at 6.0 m/s), following manufacturers recommendations. The samples were then spun at 10,000×*g* for 10 min to remove beads. The samples were incubated on a rotator for an hour and a half at 4°C with 50 μL Qiagen NiNTA resin. The samples were washed three times in His wash buffer, spinning down for 5 min at 9210×*g* and eluted in 75 μL using His Elution buffer (50 mM NaH_2_PO_4_, 300 mM NaCl, 250 mM Imidazole, pH 8.0).

### Cell‐free glycosylation (TiOP)

TiOP was conducted in S30 buffer with 0.1% n‐dodecyl‐β‐d‐maltopyranoside (DDM; Thermo Scientific) and 10 mM MnCl_2_ (Across Organics). A constant volume of lysed acceptor protein and glycan (Tables S2, S3 and S6) was mixed to varying volumes of lysed OSTs ranging from 100 μL to 500 μL for a total reaction volume of 1 mL. Cell‐free glycosylation was conducted at 30°C with gentle agitation overnight. Afterwards, the samples were centrifuged at 12,000×*g* for 10 min to remove reaction debris. The samples were then incubated for an hour and a half in 50 μL Ni‐NTA resin (Qiagen) at 4°C to pull down acceptor protein. After washing samples with His Wash buffer, the samples were eluted using 75 μL His Elution buffer.

### SDS‐PAGE and Western blot analyses

Samples were prepared in 4× LDS buffer (Invitrogen). Following incubation at 80°C for 10 min, 10 μL of sample and 5 μL Pageruler Plus protein prestained ladder (Thermo Scientific) were loaded into 10 or 12 well NuPAGE SDS 4–12% Bis‐Tris polyacrylamide gels (Invitrogen) and run in 3‐(N‐morpholino) propanesulphonic acid (MOPS; Invitrogen) buffer at 180 V for 80 min on ice. An Iblot 2 (Invitrogen) was used for protein transfer to a nitrocellulose membrane (Invitrogen), following manufacturer's instructions. Blots that were probed with antibodies were blocked with Phosphate Buffer Saline (PBS) Tween (0.1%) with 5% Milk (Sigma) for a minimum of 20 minutes. Where lectins were used, the 5% milk was omitted (Table S4). Primary antibody incubation was for 1 h at room temperature, for dilutions and antibodies used, see Table S4. Post‐incubation, the membrane was washed three times for 5 min each, in PBS‐Tween (0.1%). The samples were incubated with secondary antibodies Goat Anti Rabit 800 nm and Goat anti Mouse 680 nm (Odyssey), diluted to 1:10,000 in PBS‐Tween (0.1%) for 30 min, followed by three 5‐min washes in PBS‐Tween (0.1%). The membrane was incubated in PBS for 5 minutes prior to viewing on a LI‐COR (Odyssey) device. For western blot reactions green bands (800 nm) represent glycan or glycoprotein while the red band (680 nm) represents acceptor protein.

### Determination of the expression of OSTs

OSTs that failed to generate any glycosylation products (*M. schaedleri*, *F. sinusarabici*, *H. pullorum* PglB2 and Ca. *latescibacterium*) were assayed for expression. Overnight cultures grown for 16–18 h were diluted 1:50 in 2YPTG at 30°C, 180 rpm for 4–5 h until OD_600_ 0.6. PglB expression was induced by the addition of 1 mM IPTG and incubation continued overnight (18–20 h). Cells were pelleted at 3200×*g* for 15 min at 4°C and resuspended in Lysis buffer containing 1% DDM, lysed in Matrix B tubes by Fast Prep‐24 homogeniser (5 × 30 s at 6.0 m/s) (MP Biomedicals) and centrifuged at 14,000×*g* at 4°C for 10 min. His purification was performed under native conditions (QIA expressionist, QIAGEN) as follows: Supernatant was incubated with 50 μL NiNTA agarose (QIAGEN) for 1 h at 4°C on a roller and added to Pierce Spin Columns (P120100, Thermo Fisher Scientific), washed with 5 × 0.5 mL Wash buffer, and eluted with 50 μL Elution buffer containing 250 mM Imidazole. Fifteen μL was run by SDS‐PAGE, stained with imperial stain for 1 h and visualised on a LICOR at 680 nm.

### Antibiotics

Antibiotics were used at the following concentrations: ampicillin 100 μg/mL, kanamycin 50 μg/mL, spectinomycin 50 μg/mL and chloramphenicol 25 μg/mL.

### Computational tools

Amino acid sequence alignment was conducted using ClustaL Omega with default parameters. The PglB alignments were visualised in Geneious software. Phylogenetic tree was generated using MEGAX (Tamura et al., 2021) using the maximum likelihood method and the Tamura‐Nei model (Tamura & Nei, 1993). Phylogenetic tree bootstrap tested with 100 replications (Felsenstein, 1985). The phylogenetic tree was visualised in iTOL (Letunic & Bork, 2019).

### Statistical analysis

Densitometry results were calculated using ImageJ (Schindelin et al., 2012, 2015). For duplicate and triplicate experiments, statistical significance was calculated using one‐way ANOVA t test, a *p* value of ≤0.01 was considered statistically significant. For calculation of percentage difference in glycosylation to *C. jejuni*, densitometry values were used (Tables S7–S15) with the following formulae % difference to *C. jejuni* PglB glycosylation = (((nPglB – *C. jejuni* PglB)/*C. jejuni* PglB) × 100 + 100), where *n* = the various PglBs being tested. Errors bars were based on standard deviation.

## RESULTS

### Development and optimisation of a simplified cell‐free glycosylation assay

**FIGURE 3:**
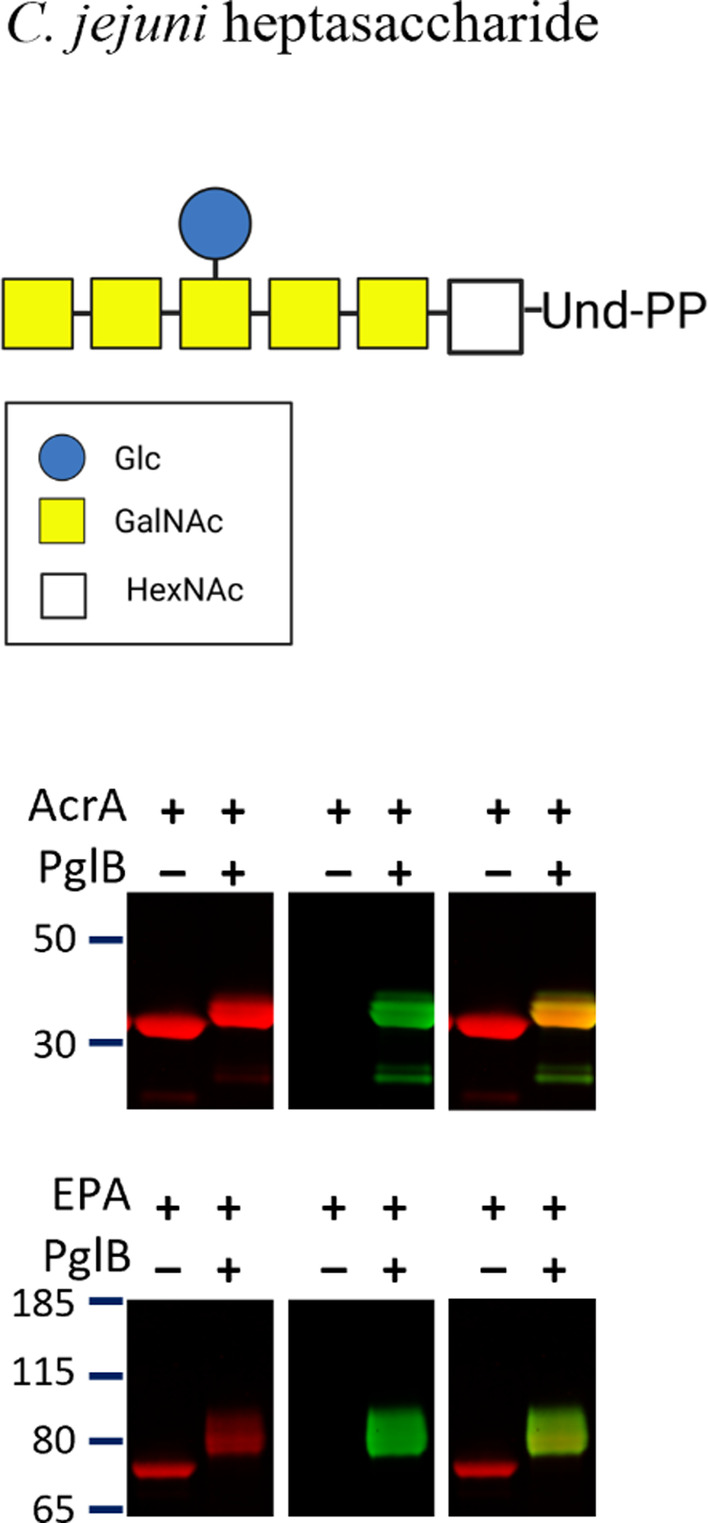
Initial TiOP assay screening. *Campylobacter jejuni* PglBs ability to glycosylate *Campylobacter* heptasaccharide to acceptor proteins AcrA or EPA. *C. jejuni* heptasaccharide with the red channel as mouse anti‐His tag and the green channel as SBA lectin.

To simplify the CFGpS process, we initially utilised the *C. jeuni pgl* locus in *E. coli*, co‐expressing *C. jejuni pglB* and its heptasaccharide. Despite mixing this lysate with the native *C. jejuni* AcrA acceptor protein (Kannicht et al., 2013), supplemented with 10 mM MnCl_2_ and 0.1% DDM, no glycosylation was detected (data not shown). Next, we separately expressed *C. jejuni* PglB, its heptasaccharide, AcrA and EPA acceptor protein, EPA was engineered to have 10 sequons (Marshall et al., 2018), in separate *E. coli* CLM24 cultures. After lysing the cells and mixing with 10 mM MnCl_2_ and 0.1% DDM, successful glycosylated product was observed (Figure 3).

To identify ideal TiOP running condition, numerous lysate preparation parameters were tested. For *C. jejuni* PglB and its heptasaccharide, omitting centrifugation post‐lysis resulted in better yields of glycosylated product, as judged by densitometry (Figure S1B,C). Efficiency of the reaction was further improved by dilution of the reaction components with S30 buffer (Figure S1A). A time course experiment between 1 to 48 h pinpointed optimal running conditions, revealing peak product yield at 16 h (Figure S2). Following TiOP optimisation, we evaluated the TiOP assays against a select number of glycans (Figure S3A–D) prior to testing its ability to assess glycosylation activity of a number of OSTs.

### Cell‐free glycosylation screening of OSTs

**FIGURE 4:**
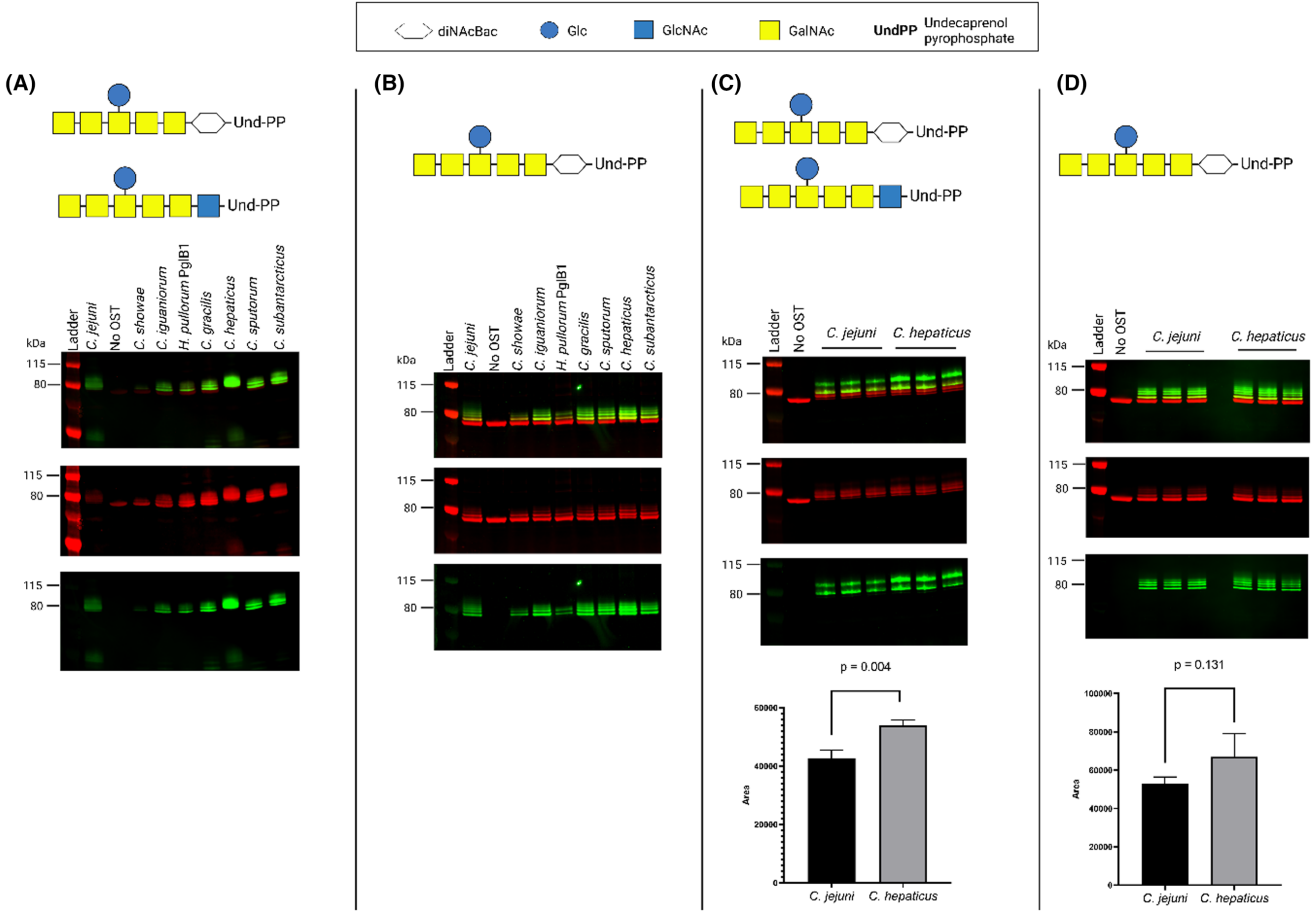
Initial Cell‐free glycosylation screening of *Campylobacter jejuni* heptasaccharide using selected PglB enzymes. (A) expressed in *E. coli* CLM24, with a mixture of DiNAcBac and GlcNAc reducing end sugars. (B) expressed in *E. coli* SDB1, with a DiNAcBac reducing end sugar. (C, D) Three biological repeats of cell‐free glycosylation of *C. jejuni* heptasaccharide expressed from *E. coli* CLM24 (C) and SDB1 (D). No OST consists of all substrates apart from OST. *C. jejuni* PglB was used as a positive control. Green bands show glycan, while red band show acceptor protein EPA.

Initial cell‐free screening of OSTs was with acceptor protein EPA, and CLM24‐expressed *C. jejuni* heptasaccharide, having a mixture of GlcNAc and DiNAcBac reducing end sugars (Tables S2 and S3; Figures S4 and S5). Seven of the eleven OSTs were capable of glycosylating EPA with the *C. jejuni* heptasaccharide (Figures S4 and S5; Table 2). Throughout the experiments, a gradual reduction in OST activity was observed with increasing volume of OST prep (Figures S4 and S5), which could be due to the components of cell lysis inhibiting the reaction. From the initial titration experiments OST volumes were chosen for further experiments (Table S5). After which TiOP was tested to compare OSTs against *C. jejuni* heptasaccharide with its native conformation of DiNAcBac as the reducing end sugar only, and one expressed in *E. coli* CLM24 with DiNAcBac/GlcNAc reducing end sugar (Figure 4).

**TABLE 2:** Summary of OST glycosylation activity tested during the study.

Glycan name	Glycan donor strain	Reducing end sugar(s)	Glycosylating OST
*Streptococcus pneumoniaee* serotype 4	*E. coli* W311O	GalNAc and GlcNAc	*C. jejuni*, *C. subantarcticus*, *C. hepaticus*, *H. pullorum PglB1*
*S*. Typhimurium B1 O antigen	*S*. Typhimurium SL3749	Gal	*C. jejuni* PglB_mut_ triple mutant S80R‐Q287P‐N311V
*Francisella tularensis* O antigen	*E. coli* DH5α	GlcNAc and Qui4NFm	*C. jejuni*, *C. iguaniorum*, *H. pullorum PglB1*, *C. gracilis*, *C. hepaticus*, *C. sputorum*, *C. subantarcticus*, *C. showae*
*E. coli* O9 antigen	*E. coli* E69	GalNAc	*C. jejuni*, *C. iguaniorum*, *H. pullorum PglB1*, *C. hepaticus*, *C. sputorum*, *C. subantarcticus*, *C. showae*
*Campylobacter jejuni* heptasaccharide	*E. coli* CLM24	DiNAcBac and GlcNAc	*C. jejuni*, *C. iguaniorum*, *H. pullorum PglB1*, *C. gracilis*, *C. hepaticus*, *C. sputorum*, *C. subantarcticus*, *C. showae*
*Campylobacter jejuni* heptasaccharide	*E. coli* SDB1	DiNAcBac	*C. jejuni*, *C. iguaniorum*, *H. pullorum PglB1*, *C. gracilis*, *C. hepaticus*, *C. sputorum*, *C. subantarcticus*, *C. showae*

Differences in DiNAcBac/GlcNAc transfer to EPA can be observed between various PglBs tested (Figure 4A), with glycosylation of both glycans by *C. hepaticus* and *C. subantarcticus* being similar with that observed for *C. jejuni* PglB, while *C. showae* transferred DiNAcBac/GlcNAc with lower efficiency than other PglBs tested. PglBs from *F. sinsusarabici*, Ca. *latesbacterium*, *M. schaedleri* and *H. pullorum* PglB2 showed no detectable transfer of the *C. jejuni* heptasaccharide with DiNAcBac/GlcNAc reducing end sugars to EPA (Figure S4).

To determine glycosylation activity in the absence of a GlcNAc reducing end sugar. OSTs were tested against the *C. jejuni* heptasaccharide expressed in *E. coli* SDB1 (Figure 4B). An upward shift of EPA glycosylated product when using *C. hepaticus* PglB was observed (Figure 4B), while *C. showae* and *H. pullorum* PglB1 showed lower glycosylation efficiency (Figure 4B). Reduced glycosylation efficiency of DiNAcBac for *H. pullorum* PglB1 when compared to *C. jejuni* PglB was also observed by Jervis and colleagues (Jervis et al., 2018). Glycosylation efficiency of *C. jejuni*, *C. gracilis* and *C. subantarcticus* PglB were similar. PglBs that did not glycosylate EPA with the *C. jejuni* heptasaccharide during the titration experiment involving *E. coli* CLM24, were also tested with DiNAcBac only *C. jejuni* heptasaccharide, with no glycosylation observed (Figure S6), the OSTs were confirmed to be expressed through Coomassie staining (Figure S7). As previous experiments suggested an upward shift of glycosylated product when using *C. hepaticus* PglB (Figure 4A,B), experiments to confirm this result were repeated in triplicate (Figure 4C,D). The results showed that *C. hepaticus* PglB glycosylation activity was superior to that of *C. jejuni* PglB when the host strain assembled the *C. jejuni* heptasaccharide with a mixture reducing end sugar of DiNAcBac and GlcNAc (*p* = 0.004, Figure 4C), while activity was on par when using the native DiNAcBac reducing end sugar (*p* = 0.131, Figure 4D).

### Testing cell‐free glycosylation against other glycans and acceptor proteins

#### Glycosyltransferase ability of OSTs in coupling *Streptococcus pneumoniae* serotype 4 to EPA

**FIGURE 5:**
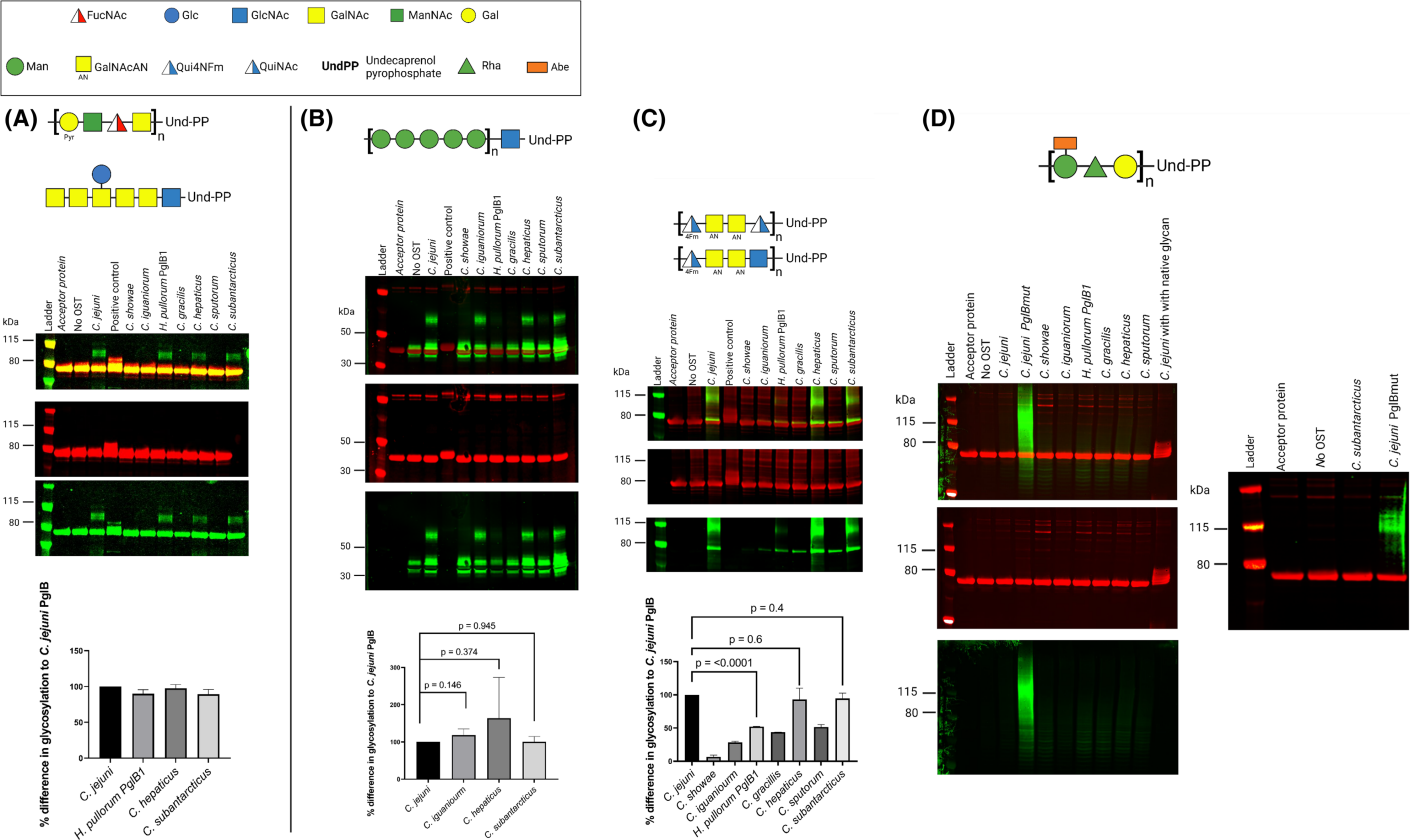
Cell‐free glycosylation screening of multiple glycans. (A) *Streptococcus pneumoniae* serotype 4 expressed from *E. coli* W311O, with GalNAc and GlcNAc reducing end sugars. (B) *E. coli* O9 antigen, expressed natively with GalNAc reducing end sugar. (C) *Francisella* O antigen expressed in *E. coli* DH5alpha (WecA and Waal +ve) with GlcNAc and Qui4NFm reducing end sugar. (D) *S*. Typhimurium B1 O antigen expressed natively with Gal reducing end sugar. *Campylobacter jejuni* PglB_mut_ has a triple amino‐acid substitution mutant S80R‐Q287P‐N311V. No OST consists of all substrates apart from OST. *C. jejuni* PglB with *C. jejuni* heptasaccharide expressed in *E. coli* CLM24 was used as a positive control. Acceptor protein column, consists of acceptor protein only, and no OST or glycan. Experiments between (A, C and D) were conducted in duplicates, while B was conducted as triplicate. Bar charts are based on % difference in glycosylation activity of other OSTs to *C. jejuni* PglB. Green bands represent glycan while red bands acceptor protein.

*Campylobacter jejuni*, *C. hepaticus*, *C. subantarcticus* and *H. pylori* PglB1 OSTs glycosylate EPA with *S. pneumoniae* serotype 4, which can be observed by a green band above 80 kDa (Figure 5A, Figure S11). Densitometry results indicated no difference in glycosylation activity between the PglBs, with p values of 0.131, 0.589 and 0.152 for *H. pylori* PglB1, *C. hepaticus*, *C. subantarcticus*, respectively, against *C. jejuni* PglB (Figure 5A). PglBs from *C. showae*, *C. iguaniorum*, *C. gracilis*, *C. sputorum* did not transfer *S. pneumoniae* serotype 4 to EPA (Figure 5A), alongside other tested OSTs (Figure S8).

#### Glycosyltransferase ability of OSTs in coupling *E. coli* O9 antigen to AcrA

A different acceptor protein to EPA, AcrA, was used for this assay, which consisted of two sequons as opposed to the engineered 10 found in EPA (Marshall et al., 2018). This was done to determine flexibility of the assay and to test the ability for OSTs to transfer the *E. coli* O9 antigen to AcrA. *C. jejuni*_,_ *C. iguaniorum*, *C. hepaticus* and *C. subantarcticus* PglBs were readily able to transfer O9 antigen to AcrA, with PglBs having no difference in glycosylation activity between each other (Figure 5B, Figure S12). Low intensity glycan bands were observed for PglBs *H. pullorum* PglB1, *C. sputorum*, *C. gracilis* and *C. showae*, suggesting a limited ability to transfer O9 antigen to AcrA (Figure 5B, Figure S12).

#### Glycosyltransferase ability of OSTs in coupling *F. tularensis* O (FTO) antigen to EPA

*Francisella tularensis* O (FTO) antigen was expressed in *E. coli* DH5α. The glycans expressed in DH5α (with GlcNAc and Qui4NFm reducing end sugars) were successfully coupled to EPA by PglBs from *C. showae*, *C. iguaniorum*, *H. pullorum* PglB1, *C. gracilis*, *C. hepaticus*, *C. sputorum* and *C. subantarcticus* (Figure 5C, Figure S13). With *C. subantarcticus* and *C. hepaticus* PglBs having glycosylation activity that was on par with *C. jejuni* PglB (*p* = 0.4 and 0.6, respectively). No glycosylation was observed when testing other OSTs (Figure S10).

#### Glycosyltransferase ability of OSTs in coupling *S. Typhimurium* O‐antigen to EPA

*Salmonella Typhimurium* B1 O‐antigen was expressed natively in the *waaL* ligase knockout strain SL3749 to determine if any of the new OSTs were capable of transferring glycans with a galactose (Gal) reducing end sugar. Native *C. jejuni* PglB is unable to transfer this glycan, but a triple amino‐acid substitution mutant S80R‐Q287P‐N311V can do so (Ihssen et al., 2015). The mutant PglB (PglB_mut_) was expressed in *E. coli* SDB1 cells and used as a positive control. *C. jejuni* PglB_mut_ transferred *S*. Typhimurium O‐antigen to EPA as previously observed by Ihssen and colleagues (Ihssen et al., 2015), which can be seen by a bright green smear (Figure 5D). Glycosylation did not occur with other OSTs tested, as the images observed are comparable to the negative control (‘No OST’) used in the experiment (Figures 5D and S9).

### Testing glycosylation activity using PGCT with *E. coli* cells

*Campylobacter hepaticus* PglB glycosylation activity was tested using PGCT. *C. hepaticus* PglB was chosen for PGCT experimentation, as it seemed to transfer *C. jejuni* heptasaccharide better than other PglBs tested during TiOP. PglBs *C. jejuni* or *C. hepaticus* were cloned into the expression vector pEXT21 and expressed in *E. coli* SDB1 with EPA acceptor protein and recombinant *C. jejuni* heptasaccharide.

**FIGURE 6:**
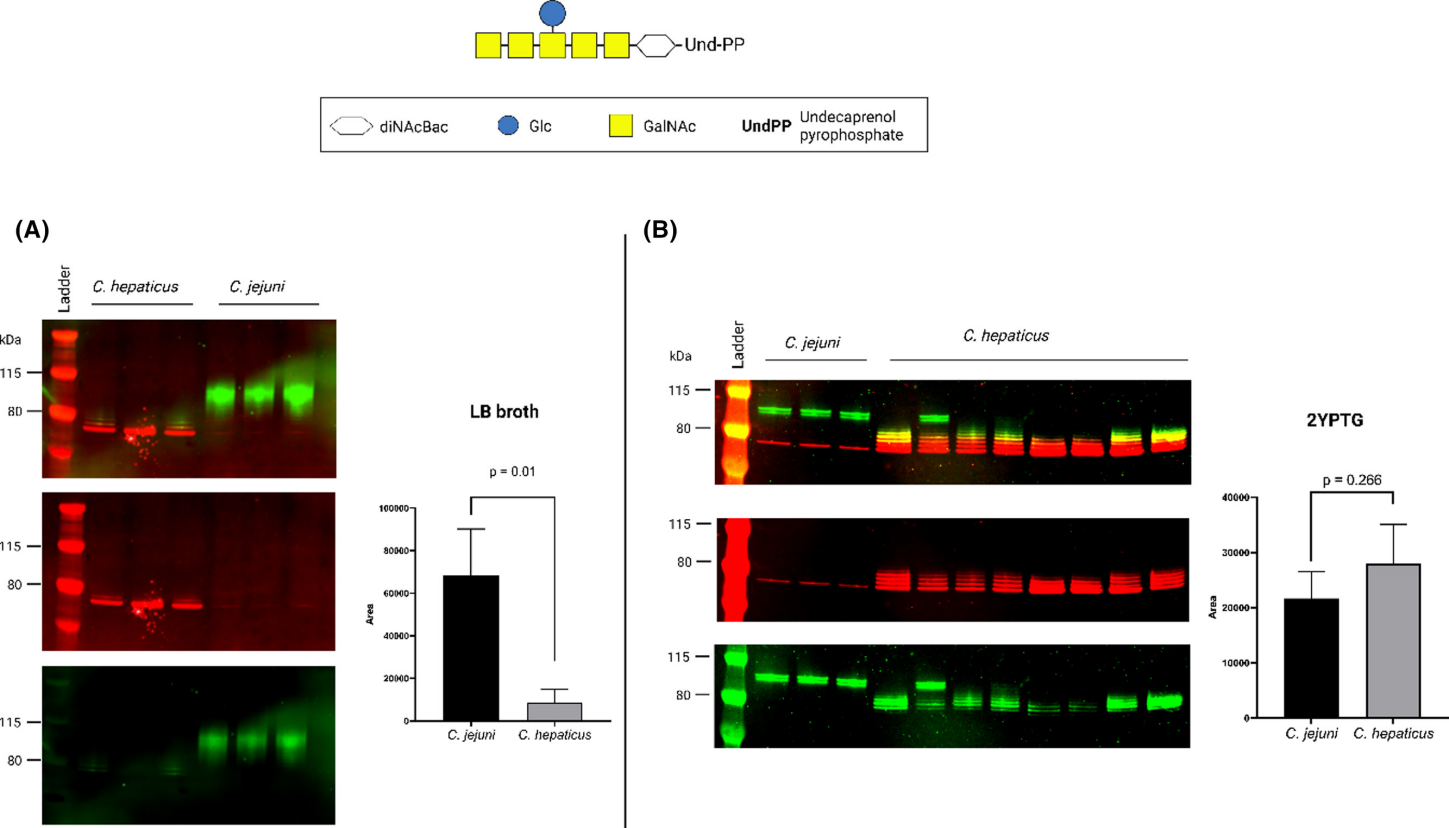
Comparing *Campylobacter jejuni* and *Campylobacter hepaticus* PglB transfer ability of *C. jejuni* heptasaccharide to EPA utilising PGCT with *E. coli* SDB1. *C. jejuni* heptasaccharide with a DiNAcBac and GlcNac reducing end sugar was tested. (A) Live *E. coli* glycosylation in LB broth and (B) live *E. coli* glycosylation in 2YPTG media. Densitometry readings are based on the first three replicates to avoid introducing biases in selection. Green bands are glycan, while red bands are acceptor protein.

Less glycosylated product was observed for *C. hepaticus* PglB than that observed when using the cell‐free system (TiOP, Figure 6A). To determine if the media used for PGCT effected *C. hepaticus* glycosylation efficiency, PGCT was conducted in 2YPTG media. PglB *C. hepaticus* glycosylation efficiency increased while using 2YPTG media (Figure 6B) when compared to LB broth (Figure 6A). The densitometry results when considering the first three replicates indicated no statistical difference in glycosylation activity (*p* = 0.266), although *C. jejuni* PglB efficiency is still higher as more of the EPA sequons have been occupied by the non‐polymerised *Campylobacter* heptasaccharide glycan, as seen by the higher molecular weight, indicative of greater glycosylation site occupancy, of all three replicates for *C. jejuni* PglB as opposed to one for *C. hepaticus* PglB (Figure 6B). There was considerable variation between *C. hepaticus* PglB biological replicates (Figure 6B). The variations could be due to the lack of optimisation of the PGCT reaction with regards to *C. hepaticus* PglB, as opposed to the assay being more optimised for the *C. jejuni* PglB. The result demonstrates the complex nature of the intracellular glycosylation system.

## DISCUSSION

Previous reports studying bacterial glycosylation used purified or chemically synthesised reaction components to produce glycosylated proteins. Jaroentomeechai et al. developed CFGpS, a ‘One Pot’ in vitro transcription, translation and glycosylation assay that expressed the glycan and OST in bacterial cells, lysed the cells, cleared pre‐existing transcription–translation complexes and added a buffer that contained all the molecular components needed to make RNA transcript and protein (Jaroentomeechai et al., 2018). To that end, rather than use transcription–translation for the acceptor protein in vitro, we decided to express all the reaction components separately in bacteria (glycan, protein and OST) and then combine them to see if glycosylation could be achieved with a less complex reaction set‐up, simplifying the process to give a ‘yes’, ‘no’; result.

**FIGURE 7:**
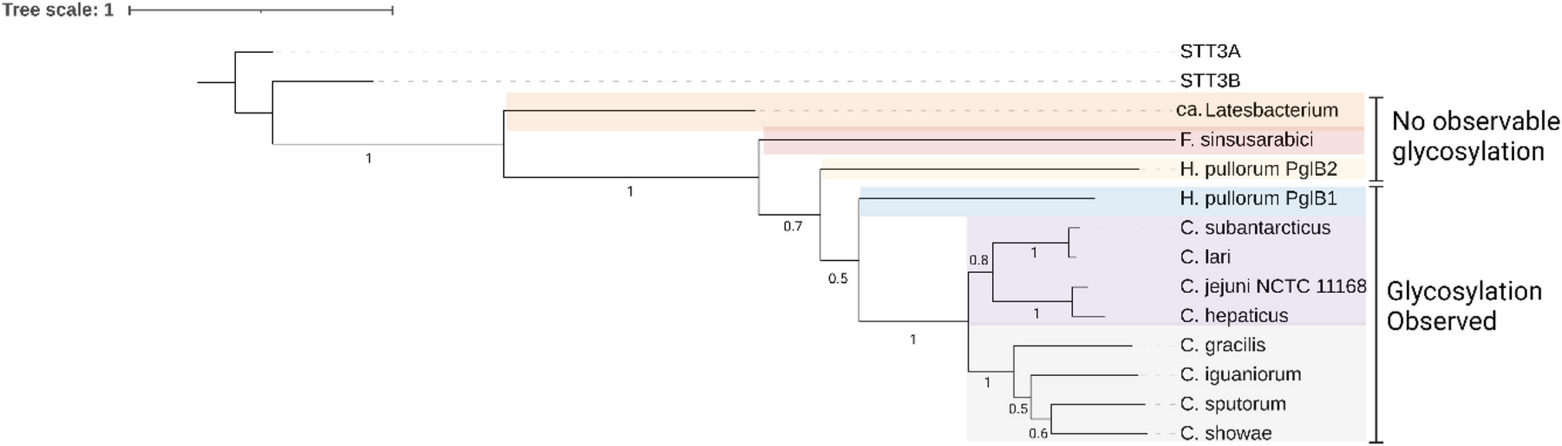
PglB Phylogenetic tree. PglB amino acid sequences were used with MEGA11 (Tamura et al., 2021). The Maximum likelihood and Tamura‐Nei model were used (Tamura & Nei, 1993), bootstrapped values are shown as numerical text (Edgar, 2004) *Campylobacter jejuni*, *Campylobacter lari*, STT3A and STT3B have been added as points of reference. Coloured squared boxes represent clades.

In this study, the investigation of glycosylating activity of diverse OSTs revealed that OSTs ranging between amino acid sequence similarity of 30–84% to *C. jejuni* PglB, successfully coupled *C. jejuni* heptasaccharide to EPA (Figure 2), while those from a different common ancestor in the case of Ca. *latesbacterium*, *F. sinusarabici* and *M. schaedleri* did not transfer any glycan tested, having lower amino acid sequence similarity to *C. jejuni* PglB (11–19%). Interestingly, PglBs from *C. sputorum*, *C. gracilis*, *C. iguaniorum* and *C. showae* did not transfer *S. pneumoniae* serotype 4 capsular polysaccharides to EPA unlike other OSTs belonging to the *C. jejuni* genus and *H. pullorum* PglB1. These findings could help future screening of OSTs against various glycans. As based on the findings it would be better, to prioritise PglBs that exhibit higher similarity to *C. jejuni* PglB for the glycans tested. While for *S. pneumoniae* serotype 4, it seems PglBs whose phylogenies are between *H. pullorum* PglB1 and the *C. jejuni* PglB (purple‐coloured clade), should be prioritised for further investigation (Figure 7). Considering any known conserved amino acid residues vital for PglB function (Barre et al., 2017).

*H. pullorum* PglB1 (Figure 7, blue clade) had the ability to transfer many of the glycans tested, unlike PglB2 (Figure S4 and Table 2); which is distinct enough to *H. pullorum* PglB1 to be placed within a different clade to PglB2 (Figure 7, yellow clade). The PglB1 clade is located closer to the *C. jejuni* genus and has a higher amino acid sequence similarity to *C. jejuni* PglB of 30%, as opposed to 19% for *H. pullorum* PglB2 (Figure 2). Natively, *H. pullorum* PglB1 has been shown to transfer a linear pentasaccharide with a HexNAc reducing end sugar (Jervis et al., 2018) and has also been shown to transfer a DiNAcBac reducing end heptasaccharide in *E. coli* (Jervis et al., 2010, 2018). The function of *H. pullorum* PglB2 is unknown and has been hypothesised to be associated with transferring diNAcBac reducing end sugar, due to the presence of the *pglFED* homologues in *H. pullorum* (Jervis et al., 2010, 2018), although in the current study *H. pullorum* PglB2 did not transfer *C. jejuni* heptasaccharide with a diNAcBac reducing end sugar. We demonstrated that TiOP was able to rapidly test OSTs with glycan donors prepared directly from numerous bacteria. The ability to prepare and use glycan lysate from their native bacteria removes the requirement to clone and recombinantly express glycans to test OST compatibility. It would be interesting to test the ability of OSTs to transfer their native glycan and those closely related, which may aid in increasing the spectrum of glycans that can be transferred by OSTs. Testing native *H. pullorum* glycan to determine whether *H. pullorum* PglB2 plays a role in protein glycosylation would be interesting, as the enzyme has all the essential residues required for PglB glycosyltransferase activity (Jaroentomeechai et al., 2018).

The most distantly related PglB to *C. jejuni* was Ca. *latescibacterium* PglB. BLASTN search of Ca. *Latesbacterium pglb* against the non‐redundant NCBI database identified that the closest sequenced genome was Ca. *latescibacteria* bacterium DG_63 (Accession number ASM130290v1). The genes surrounding the Ca. *latesbacterium* PglB orthologue all consisted of genes of unknown function. Ca. *latesbacterium* PglB has higher similarity to STT3A (56.1%) and STT3B (54.4%) domains found in eukaryotes than *C. jejuni* PglB and others tested in this study (29.1–37.1%). Therefore, it would be interesting to test Ca. *latesbacterium* PglB against a glycan similar to that found in eukaryotes, i.e. a high mannose glycan with a GlcNAc reducing end sugar (Glc3Man9GlcNAc2) (Dell et al., 2010).

Cross comparison between experiments is difficult due to uncertainty in the amount of glycan expressed for each polysaccharide tested during the study. For the current study, a titration of OSTs was done (Figures S4 and S5) and an ideal volume was chosen for testing glycosyltransferase activity. Ideally, OST, carrier and glycan would be quantified, although the study shows that TiOP can be utilised as a crude rapid screening method for variables relating to PGCT. The cell‐free assay could potentially be able to distinguish between efficiencies in glycosylation reactions and thus could be used to rapidly test variables, such as glycans, and OSTs prior to testing in a cell‐based assay. For cross comparison between acceptor proteins, the number of sequons present would have to be considered prior to comparison, as the intensity of the bands/number of bands observed via densitometry analysis could result in bias. In terms of number of bands observed for future studies, with better separation and granularity of western blot images, enumeration of the number of polymerised glycan coupled to acceptor protein sequons could help in assessing glycosylation efficiency for reaction components. Furthermore, once OSTs of interest are selected, TiOP could potentially be used to test ideal amino acid residue(s) for an acceptor protein sequon. As an example, for PglB a threonine instead of a serine within D/E‐X‐N‐X‐S/T, where X can be any amino acid residue other than proline; is known to improve PglB catalytic efficiency (Gerber et al., 2013); tests could be conducted on the effects of such changes on the catalytic efficiency of other PglBs besides *C. lari*.

TiOP and PGCT showed that *C. hepaticus* PglB transfers *C. jejuni* heptasaccharide to EPA (Figure 6). Unsurprisingly, recent studies have demonstrated similarities in the structure of the *C. hepaticus* heptasaccharide to that of *C. jejuni* (McDonald et al., 2023) and is a likely explanation for the similar glycosylation efficiency for the two PglBs. PGCT with *C. hepaticus* PglB showed a dramatic improvement in glycosylation efficiency when changing culture media (Figure 6). Moreover, there were varying results between the multiple repeats for *C. hepaticus* PglB tested during the study (Figure 6B), which may be an indication of a lack of optimisation for the biological system. Further optimisation is likely to improve PGCT outcomes when using other PglBs. Optimising glycosylation conditions for each variable tested within the study in a live cell system would be laborious and could lead to false negatives if testing a large selection of OSTs. For PGCT assay, expression of a number of genes involved in glycoprotein production in the periplasm needs to be optimised, as the glycan needs to be synthesised in the cytoplasm and flipped to the periplasm where the PglB enzyme and acceptor protein have to be present for glycosylation to occur (Figure 1A). The cell‐free assay used in the study, where proteins and glycans were expressed independently and glycosylation occurred outside the periplasm of the cell (Figure 1B), bypasses the need for the concerted actions of three components in a single cell, thus is useful for ascertaining compatibility of OSTs, glycans and acceptor proteins. The streamlined TiOP developed in this study did not achieve the same level of glycosylation as shown in the original method using transcription–translation, but the simplification allows for a much faster testing process for glycosylation. Once glycosylation is confirmed then PGCT can be optimised for downstream applications such as glycoconjugate vaccine production.

## CONCLUSION

This study shows the simplification of an existing cell‐free glycosylation assay to rapidly screen OST, glycan and carrier protein combinations for efficient glycosylation. Despite lacking quantifiable accuracy, the assay can quickly provide answers as to which combination of glycan, carrier protein and OST can work in combination, thus potentially accelerating glycoconjugate vaccine development. The assay was used to test multiple OSTs for glycotransferase activity with different protein acceptor/glycan combinations. PglBs from *C. showae*, *C. iguaniorum*, *H. pullorum PglB1*, *C. gracilis*, *C. hepaticus*, *C. sputorum* and *C. subantarcticus* could transfer several glycans. Transfer activity of PglBs from *C. iguaniorum*, *C. gracilis*, *C. hepaticus* and *C. subantarcticus* was not previously demonstrated in a cell‐free system. High transfer activity was observed for PglBs from *C. hepaticus*, *C. subantarcticus* and *C. jejuni* PglB. With *C. hepaticus* and *C. subantarcticus* having 84% and 56% amino acid similarity, respectively, to *C. jejuni* PglB. The cell‐free system is ideal to rapidly assess for functional/non‐functional output prior to optimisation with PGCT. The cell‐free system can test glycans without the need for recombinant expression. This is important for glycans with loci that are difficult to express recombinantly and is useful for screening whether a given glycan is compatible with a specific OST prior to identifying the glycan of interest i.e., prior to sequencing the genome of a bacterium. The cell‐free system described here should be a useful approach to screen PglBs including mutated variants and increase the efficiency and scope of glycan transfer and facilitate the development of glycoconjugate vaccines against several bacterial pathogens.

## AUTHOR CONTRIBUTIONS

**Burhan Lehri:** Data curation; formal analysis; investigation; writing – original draft; writing – review and editing. **Elizabeth Atkins:** Data curation; formal analysis; investigation; methodology; writing – review and editing. **Timothy A. Scott:** Investigation; writing – review and editing. **Sherif Abouelhadid:** Investigation; writing – review and editing. **Brendan W. Wren:** Supervision; writing – review and editing. **Jon Cuccui:** Conceptualization; funding acquisition; supervision; writing – review and editing.

## FUNDING INFORMATION

No funding information provided.

## CONFLICT OF INTEREST STATEMENT

The authors declare no conflict of interests.

## Supporting information


Appendix S1.
