# Supplementary material for: Investigation into the efficiency of diverse N‐linking oligosaccharyltransferases for glycoengineering using a standardised cell‐free assay
Source: Microb Biotechnol. 2024 Jun 10;17(6):e14480. doi: 10.1111/1751-7915.14480 (PMC11164674; doi:10.1111/1751-7915.14480)
Supplement: Supplementary file 1 — Appendix S1. [file MBT2-17-e14480-s001.docx]

**Supplementary**

**Table S1.** Primers for confirming presence and orientation of PglB inserts onto pEXT21 plasmid.

| **Colony PCR primer List** | Sequence | Expected product size (bp) |
| --- | --- | --- |
| Plasmid MCS_ forward primer | GCATGCCTGCAGGTCGACTC | N/A |
| *C. showae* reverse primer | CCTGCCGGACAGCATGATTGATA | 496 |
| *F. sinsusarabici* reverse primer | GATCGGCAAGTATGGCGCG | 508 |
| *C. iguaniorum* reverse primer | CGGTAGTTGCGAAGTTCTCACG | 470 |
| *H. pullorum* PglB1 reverse primer | GAGAACTCAGAGGCCAAGGCG | 801 |
| *H. pullorum* PglB2 reverse primer | CTGACCAATCAAGAGATCCGTTCG | 756 |
| *Ca. Latesbacterium* reverse primer | GACAACGGCACGTGGAACTG | 573 |
| *M. chaedleri* reverse primer | GCTGACCGAGAACGGTCGTC | 301 |
| *C. gracilis* reverse primer | CTAACATCGACATCACCGACGG | 452 |

**Table S2.** Titration determining functional volume to use for PglB; *C. showae,* *C. iguaniorum*, *H. pullorum* PglB1, *C. gracilis, C. Latesbacterium, M. Schaedleri, F. sinsusarabici*.

| **Name on gel** | **Acceptor**  **protein** | **Oligosacharyltransferase** | | **Glycan** | **Buffer** | **Cofactor** |  |
| --- | --- | --- | --- | --- | --- | --- | --- |
|  | **ExoA (µl)** | **Name** | **Volume (µl)** | **CjLLO (µl)** | **S30 (µl)** | **DDM/**  **Manganese (µl)** | **Total (µl)** |
| ***C. jejuni***  **positive control** | 20 | PglB *C. jejuni* | 75 | 100 | 795 | 10 | 1000 |
| **0 µl** | 20 | - | 0 | 100 | 870 | 10 | 1000 |
| **20 µl** | 20 | Testing OST | 20 | 100 | 850 | 10 | 1000 |
| **40 µl** | 20 | Testing OST | 40 | 100 | 830 | 10 | 1000 |
| **60 µl** | 20 | Testing OST | 60 | 100 | 810 | 10 | 1000 |
| **80 µl** | 20 | Testing OST | 80 | 100 | 790 | 10 | 1000 |
| **100 µl** | 20 | Testing OST | 100 | 100 | 770 | 10 | 1000 |
| **200 µl** | 20 | Testing OST | 200 | 100 | 670 | 10 | 1000 |
| **300 µl** | 20 | Testing OST | 300 | 100 | 570 | 10 | 1000 |
| **400 µl** | 20 | Testing OST | 400 | 100 | 470 | 10 | 1000 |
| **500 µl** | 20 | Testing OST | 500 | 100 | 370 | 10 | 1000 |

**Table S3.** Titration for determining functional volume to use for PglB; *C. sputorum, C. subantarcticus, C hepaticus*.

| **Name on gel** | **Acceptor**  **protein** | **Oligosacharyltransferase** | | **Glycan** | **Buffer** | **Cofactor** |  |
| --- | --- | --- | --- | --- | --- | --- | --- |
|  | **ExoA (µl)** | **Name** | **Volume (µl)** | **CjLLO (µl)** | **S30 (µl)** | **DDM/**  **Manganese (µl)** | **Total (µl)** |
| ***C. jejuni***  **positive control** | 20 | PglB *C. jejuni* | **75** | 100 | 795 | 10 | 1000 |
| **0 µl** | 20 | - | 0 | 100 | 870 | 10 | 1000 |
| **20 µl** | 20 | Testing OST | 20 | 100 | 850 | 10 | 1000 |
| **40 µl** | 20 | Testing OST | 40 | 100 | 830 | 10 | 1000 |
| **60 µl** | 20 | Testing OST | 60 | 100 | 810 | 10 | 1000 |
| **100 µl** | 20 | Testing OST | 100 | 100 | 770 | 10 | 1000 |
| **200 µl** | 20 | Testing OST | 200 | 100 | 670 | 10 | 1000 |
| **300 µl** | 20 | Testing OST | 300 | 100 | 570 | 10 | 1000 |

**Table S4.** Primary antibodies used for the detection of glycan and Hexa Histidine tagged acceptor protein for western blots.

| **Glycan** | **Primary antibody** |
| --- | --- |
| *S. pneumoniae capsule* serotypes 4 | Rabbit SSI Type serum 4 pneumococcus, SSI Diagnostica (Dilution 1:1000) and Mouse anti-His tag, thermofisher scientific (Dilution 1:10000) |
| *S.* Typhimurium B1 O antigen | Salmonella O antiserum Group B Factors 1, 4, 5, 12 (BD) |
| *Francisella tularensis* O antigen | Antifrancisella Tularensis LPS (monoclonal mouse AB) from Biologicals a biotechne (Dilution 1:10000) and Rabbit anti-His tag, thermofisher scientific (Dilution 1:10000) |
| *C. jejuni* Heptasachharide | Lectin SBA biotin, Vector Laboratories (Dilution 1:10000) and Mouse anti-His tag, thermofisher scientific (Dilution 1:10000) |
| *E. Coli* O9 antigen | ConA Lectin, Vector Laboratories (Dilution 1:10000) and Mouse anti-His tag, thermofisher scientific (Dilution 1:10000) |

**Table S5.** Selected volumes as deduced by titration experiment.

| **Oligosaccharyltransferase** | **Volume (µl)** |
| --- | --- |
| PglB *C. jejuni* | 75 |
| PglB *C. showae* | 100 |
| PglB *C. iguaniorum* | 40 |
| PglB *H. pullorum PglB1* | 100 |
| PglB *C. gracilis* | 100 |
| PglB *C. hepaticus* | 100 |
| PglB *C. sputorum* | 40 |
| PglB *C. subantarcticus* | 60 |
| *C. jejuni* PglB_mut_ (S80R-Q287P-N311V) | 200 |

**Table S6.** Glycan Volume used.

| **Glycan Name** | **Cell line** | **Reducing end sugar(s)** | **Volume used (µl)** |
| --- | --- | --- | --- |
| *Streptococcus pneumoniae serotype* 4 | *E. coli* W311O | GalNAc and GlcNAc | 200 |
| *S.* Typhimurium B1 O antigen | *S. typhimurium* SL3749 | GalNAc | 200 |
| *Francisella tularensis* O antigen | *E. coli* DH5alpha | GlcNAc and Qui4NFm | 200 |
| *E. coli* O9 antigen | Native | GalNAc | 400 |
| *C. jejuni* heptasaccharide | *E. coli* Clm24 | diNAcBac and GlcNAc | 100 |
| *C. jejuni* heptasaccharide | *E. coli* SDB1 | diNAcBac | 100 |


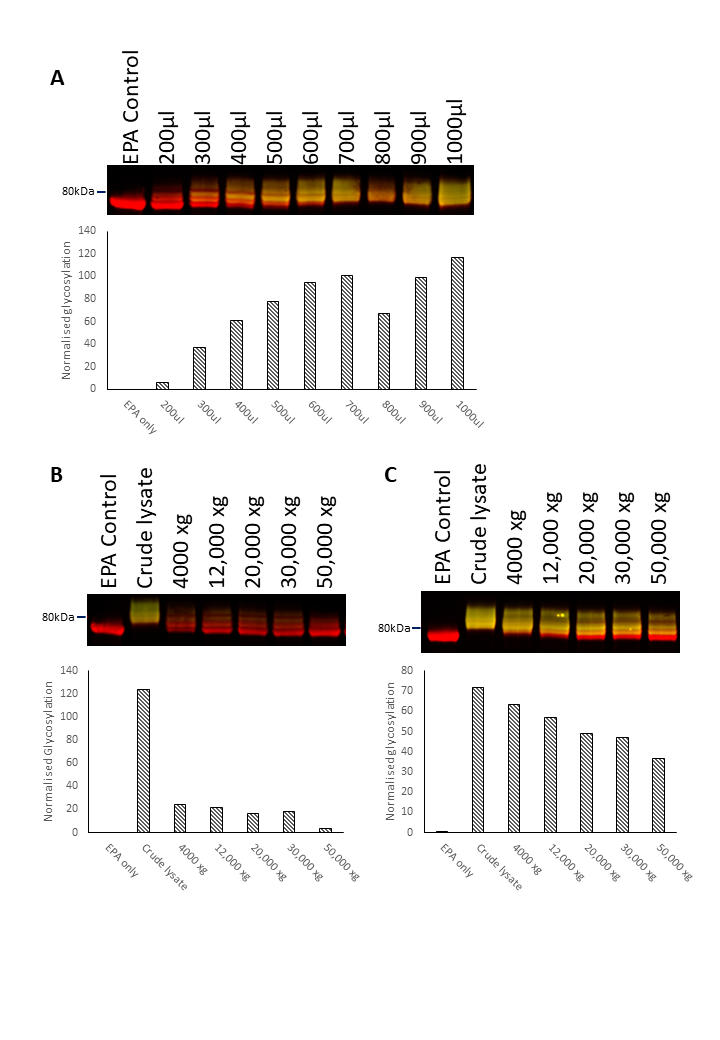


**Figure S1. Optimisation of TiOP method.** Optimisation experiments with acceptor protein EPA, *C. jejuni* PglB and *C. jejuni* heptasaccharide. All blots show mouse anti-His antibody in the red channel and SBA lectin in the green channel. Densitometry results were determined as the mean pixel intensity of a set area covering all bands in a lane in both the red and green channels, which were then normalised to set the red channel as a constant for each lane. (A) Dilution series where reactions consisting of the same volumes of lysate for donor, acceptor and PglB were progressively diluted with S30 buffer, reactions were run overnight then proteins purified. (B) Subjecting the glycan donor lysate to progressively higher centrifugation forces to remove cell debris. (C) Subjecting the PglB lysate to progressively higher centrifugation forces to remove cell debris.


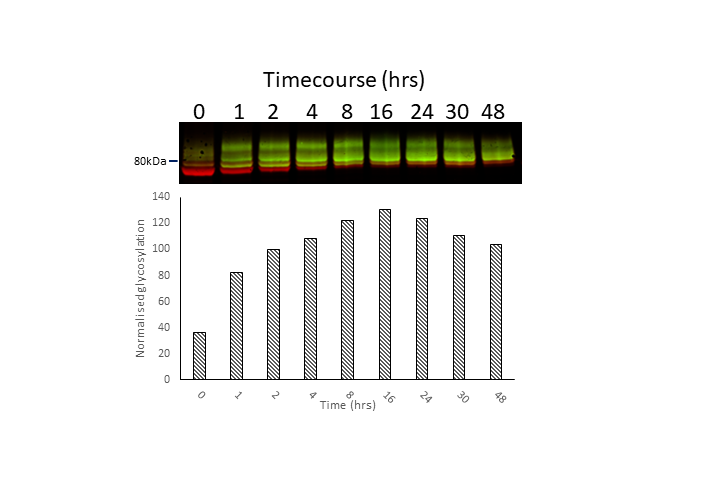


**Figure S2. Optimising TiOP reaction time**. TiOP using *C. jejuni* PglB, its heptasaccharide and acceptor protein EPA were incubated for up to 48 hours to test the efficiency of the reaction over time. Densitometry results were determined as the mean pixel intensity of a set area covering all bands in a lane in both the red and green channels, which were then normalised to set the red channel as a constant for each lane. EPA (red channel), glycan (green channel).


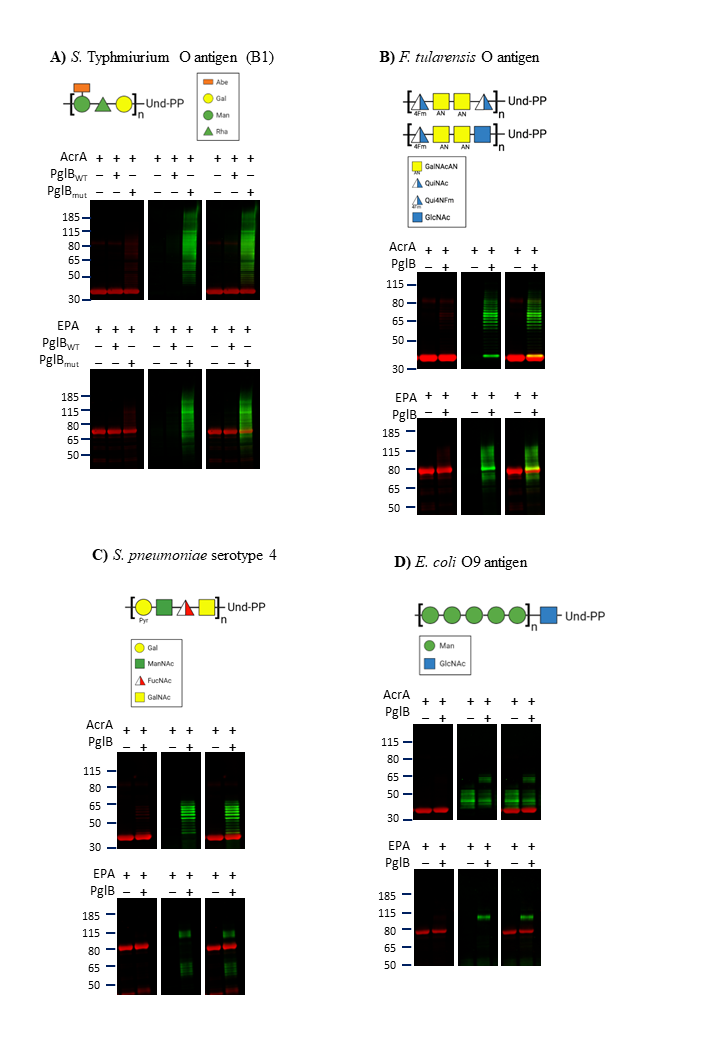


**Figure S3. Western blots showing AcrA and EPA protein glycosylation.** A range of bacterial glycans were tested with *C. jejuni* PglB or PglB_mut_ (a triple amino-acid substitution mutant S80R-Q287P-N311V) and acceptor protein AcrA or EPA. For all blots, left panel is AcrA and right panel is EPA. (A) *S. typhimurium* with the red channel as mouse anti-His tag and the green channel as rabbit O4 and O5 antisera. (B) *F. tularensis* with the red channel as rabbit anti-His tag and the green channel as mouse anti-Francisella LPS (FB11). (C) *S. pneumoniae* with the red channel as mouse anti-His tag and the green channel as rabbit Sp4 antisera. (D) *E. coli* O9 with the red channel as mouse anti-His tag and the green channel as ConA lectin.


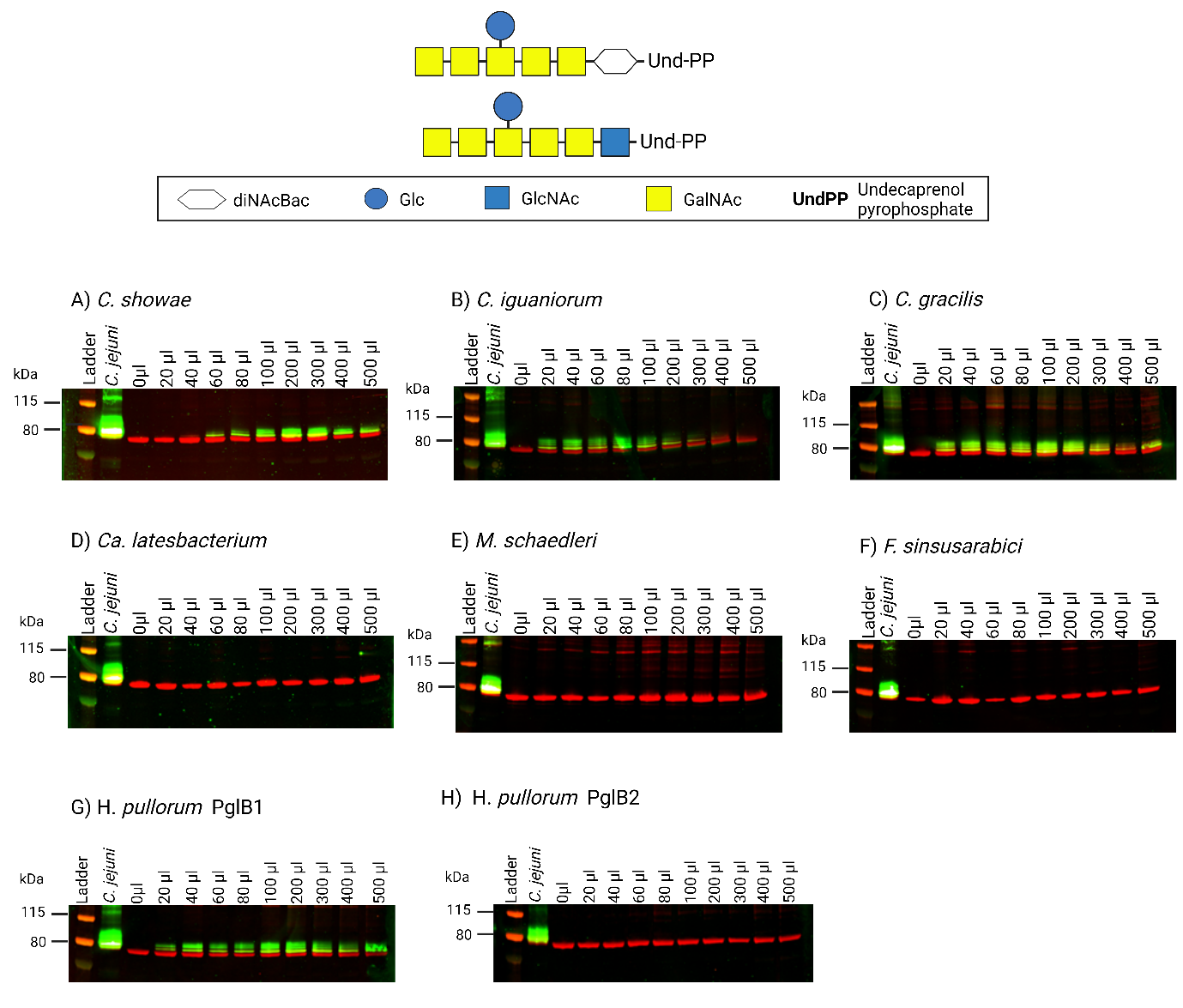


**Figure S4. Initial optimisation of cell free glycosylation.** Cell free glycosylation of *C. jejuni* heptasaccharide expressed from *E. coli* Clm24, with diNAcBac and GlcNAc reducing end sugars. Titres of PglBs; *C. showae*, *C. iguaniorum*, *C. latesbacterium, M. schaedleri,* *F. sinsusarabici*, *H. pullorum* PglB1 and *H. pullorum* PglB2 were tested. *C. jejuni* PglB was used as a positive control. Green and red bands represent glycan and protein respectively.


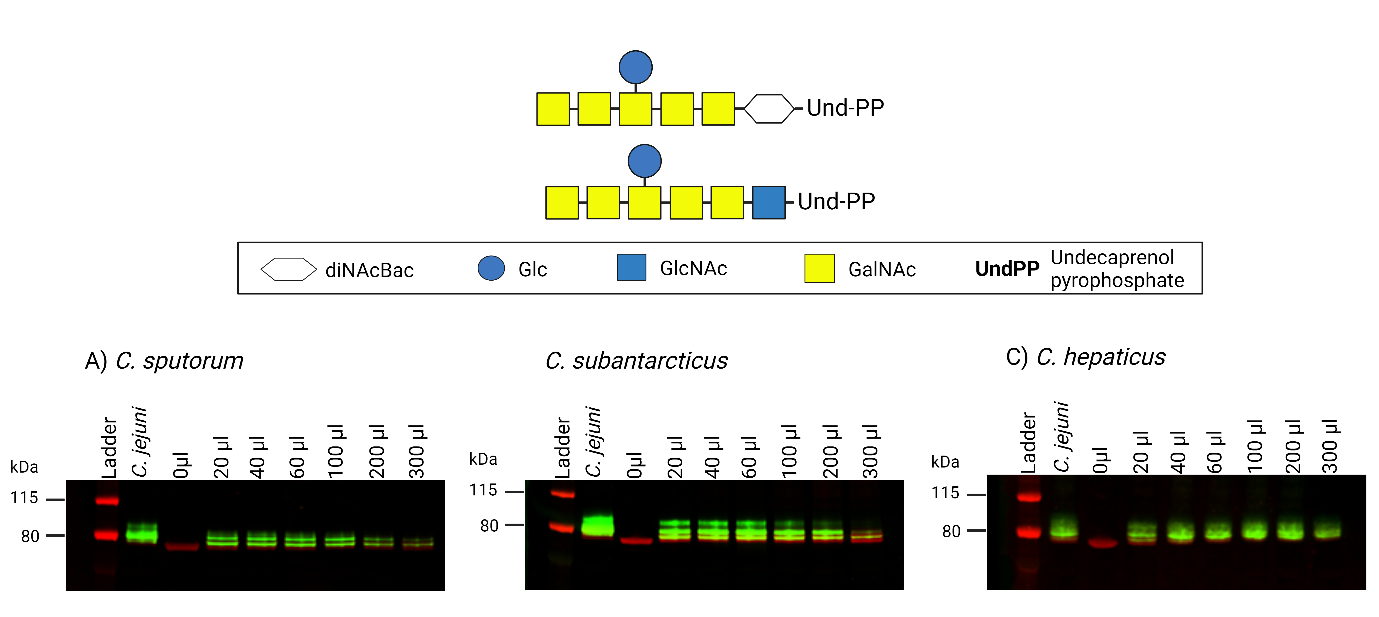


**Figure S5. Optimisation of cell free glycosylation.** Cell free glycosylation of *C. jejuni* heptasaccharide expressed from *E. coli* Clm24, with diNAcBac and GlcNAc reducing end sugars. Titres of PglBs; *C. sputorum*, *C. subantarcticus*, *C. hepaticus* were tested. *C. jejuni* PglB was used as a positive control. Green and red bands represent glycan and protein respectively.


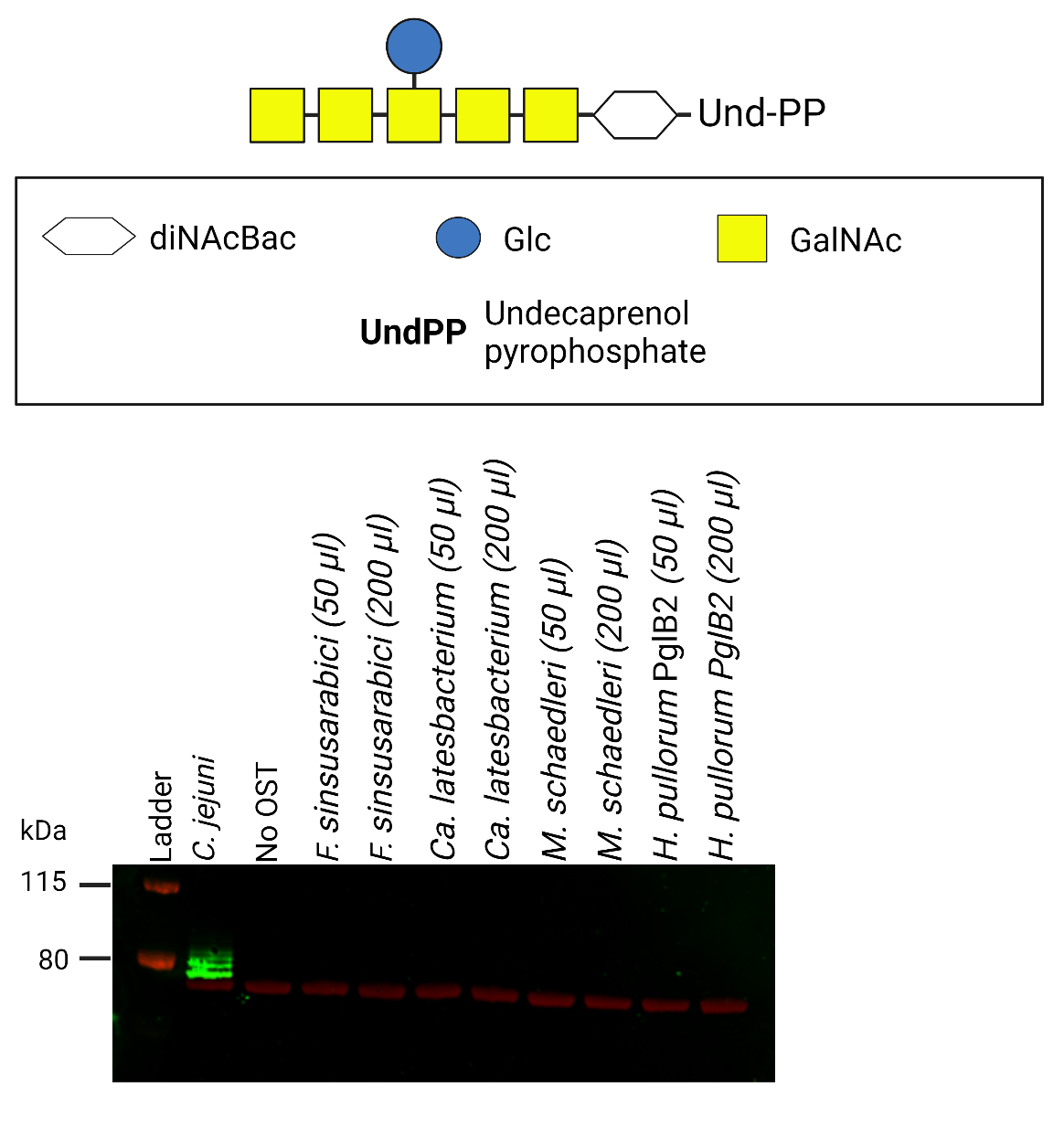


**Figure S6. Testing glycosylation of PglBs from *F. sinsusarabici, Ca. latesbacterium, M. schaederi, and H, pullorum PglB2* to diNAcBac reducing end sugar only.** Western Blot results for cell free glycosylation of *C. jejuni* heptasaccharide expressed from *E. coli* SDB1, with diNAcBac reducing end sugar. No OST represents negative control, while *C. jejuni* PglB was used as a positive control. Green and red bands represent glycan and protein respectively.


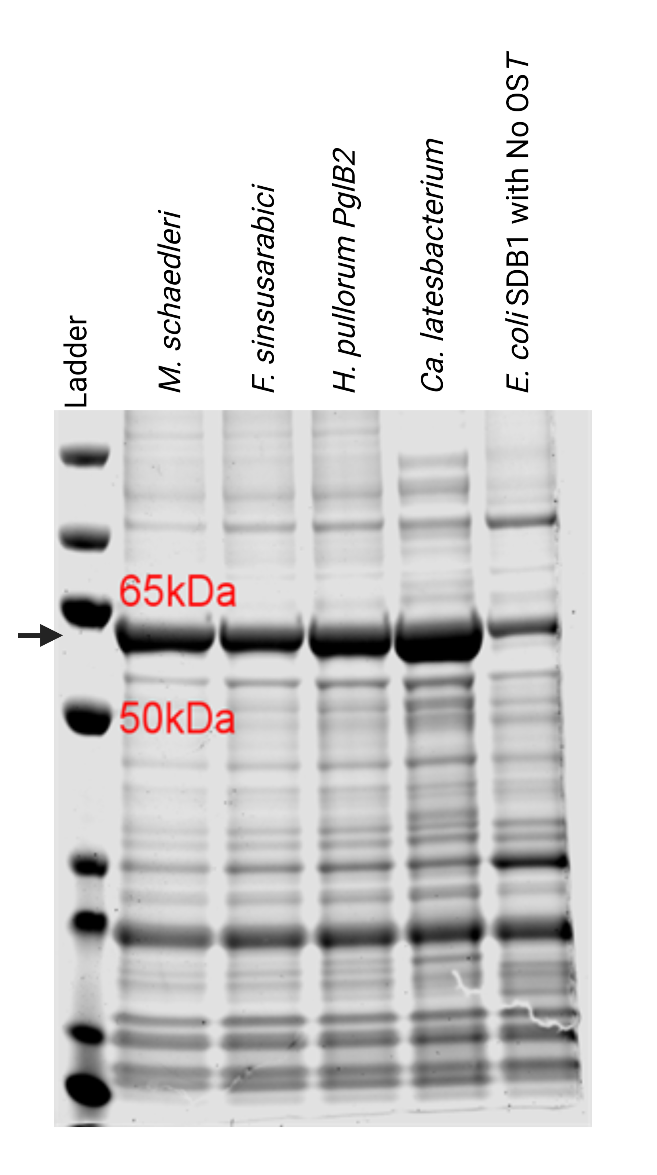


**Figure S7.** **Determining PglB expression.** Coomassie stain of PglB expressed in *E. coli* SDB1, along with *E. coli* SDB1 lysate without a *pglB* gene.


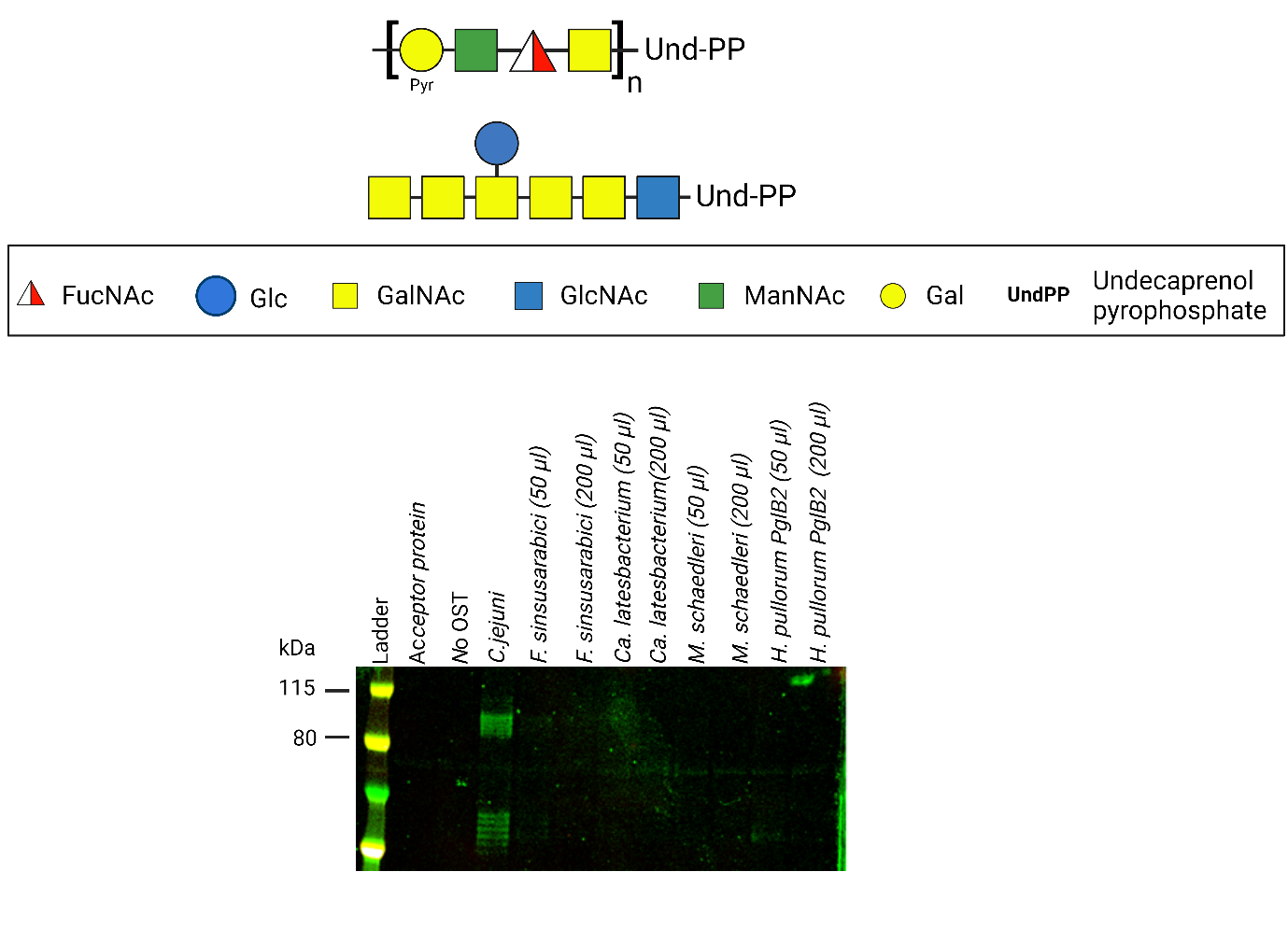


**Figure S8.** **Cell free glycosylation of *Streptococcus pneumonia serotype 4*.** *S. pneumonia serotype* 4 expressed from *E. coli* W311O, with GalNAc and GlcNAc reducing end sugars. No OST represents negative control, while *C. jejuni* PglB with *C. jejuni* heptasaccharide expressed in *E. coli* Clm24 was used as a positive control. Well labelled acceptor protein consists of acceptor protein only, Goat Anti Rabit 800 nm antibody was not added during the experiment leading to no acceptor protein band.


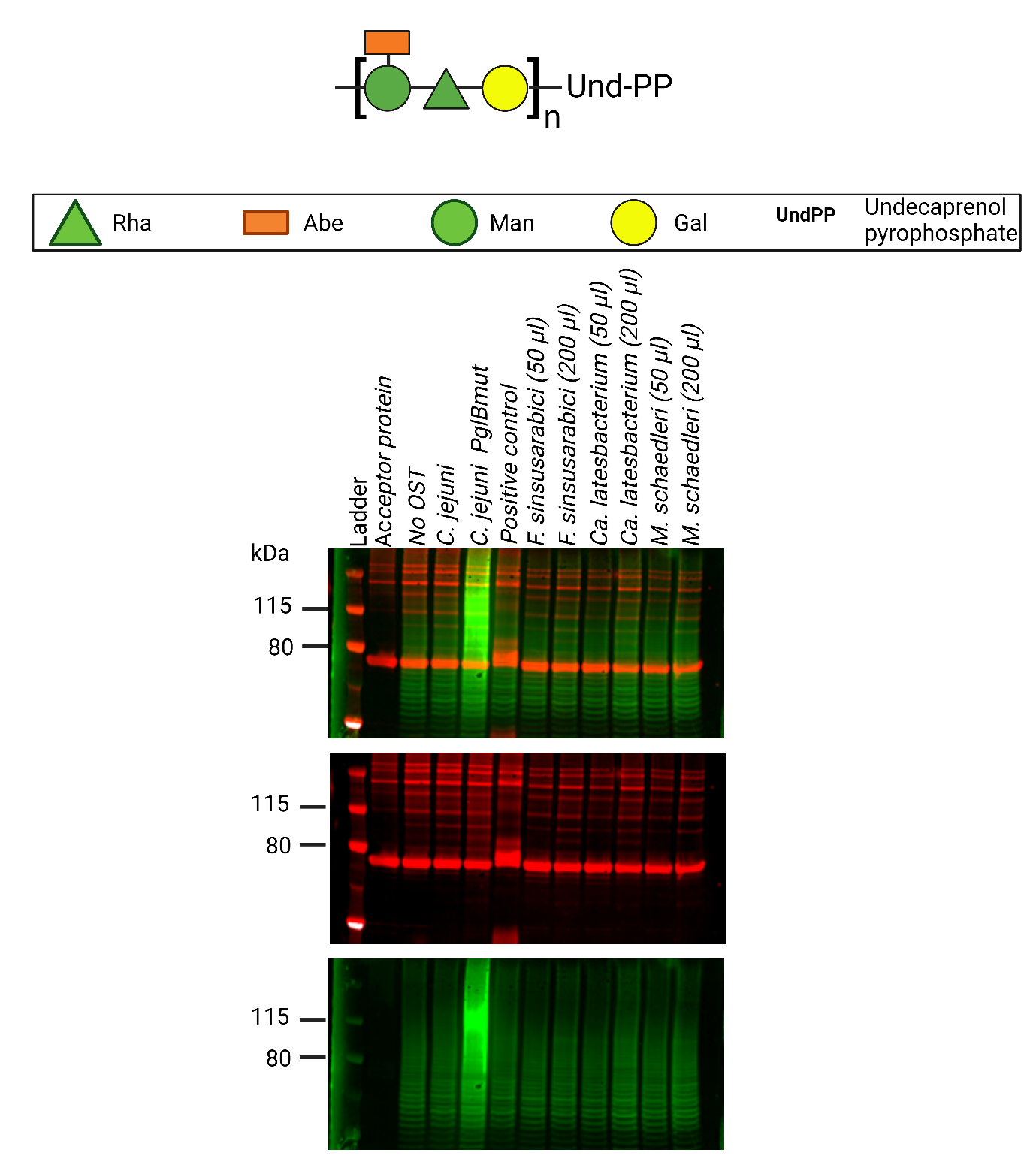


**Figure S9. Cell free glycosylation of S. Typhimurium O-antigen**. expressed natively with GalNAc reducing end sugar. No OST and acceptor protein only represents negative control, while *C. jejuni* PglB with *C. jejuni* heptasaccharide expressed in *E. coli* Clm24 was used as a positive control. Well labelled acceptor protein consists of acceptor protein only. Green and red bands represent glycan and protein respectively.


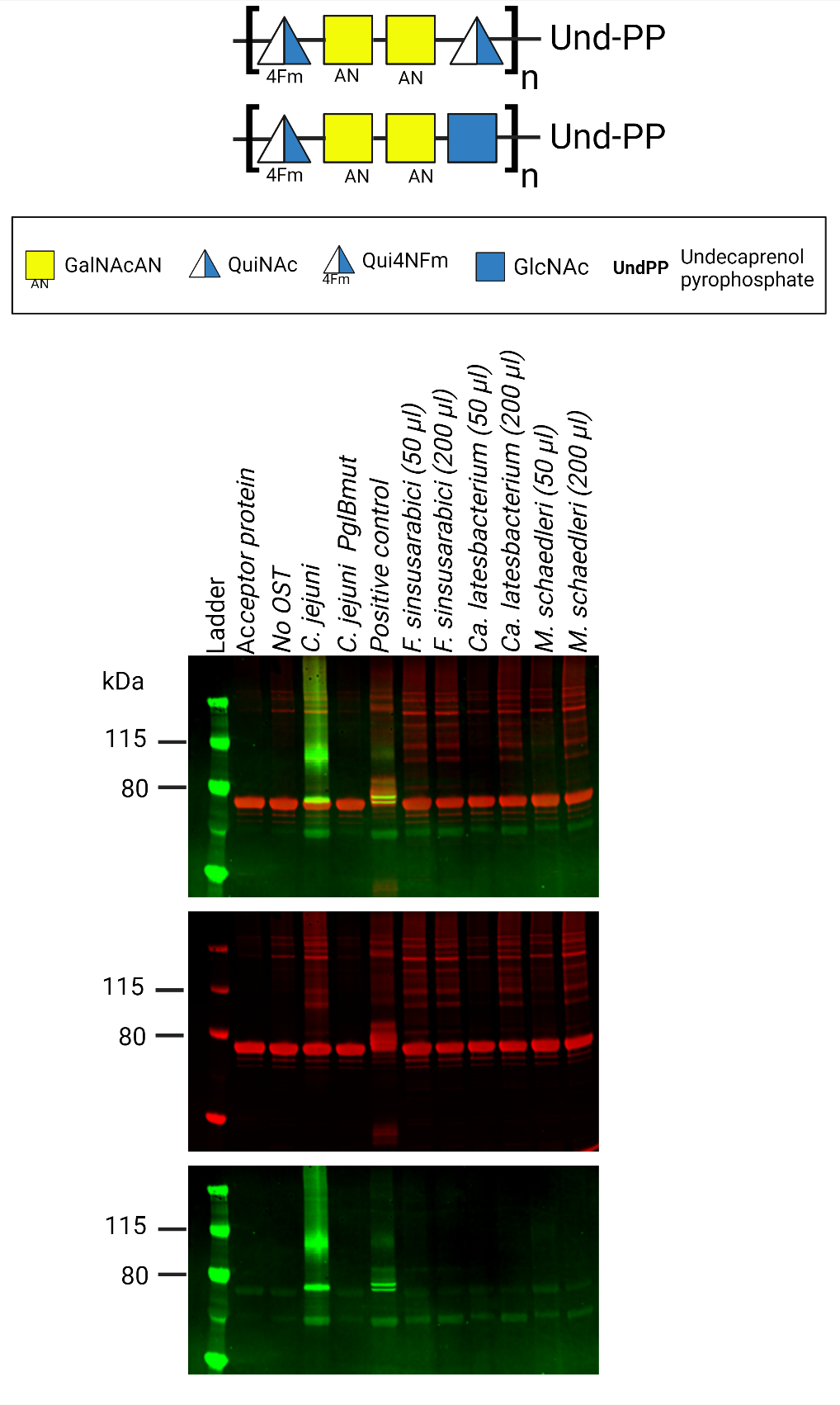


**Figure S10.** **Cell free glycosylation of *Francisella tularensis O* antigen expressed in *E. coli* DH5alpha synthesising GlcNAc and Qui4NFm reducing end sugars.** No OST and acceptor protein only represents negative control, while *C. jejuni* PglB with *C. jejuni* heptasaccharide expressed in *E. coli* Clm24 was used as a positive control. Green and red bands represent glycan and protein respectively.


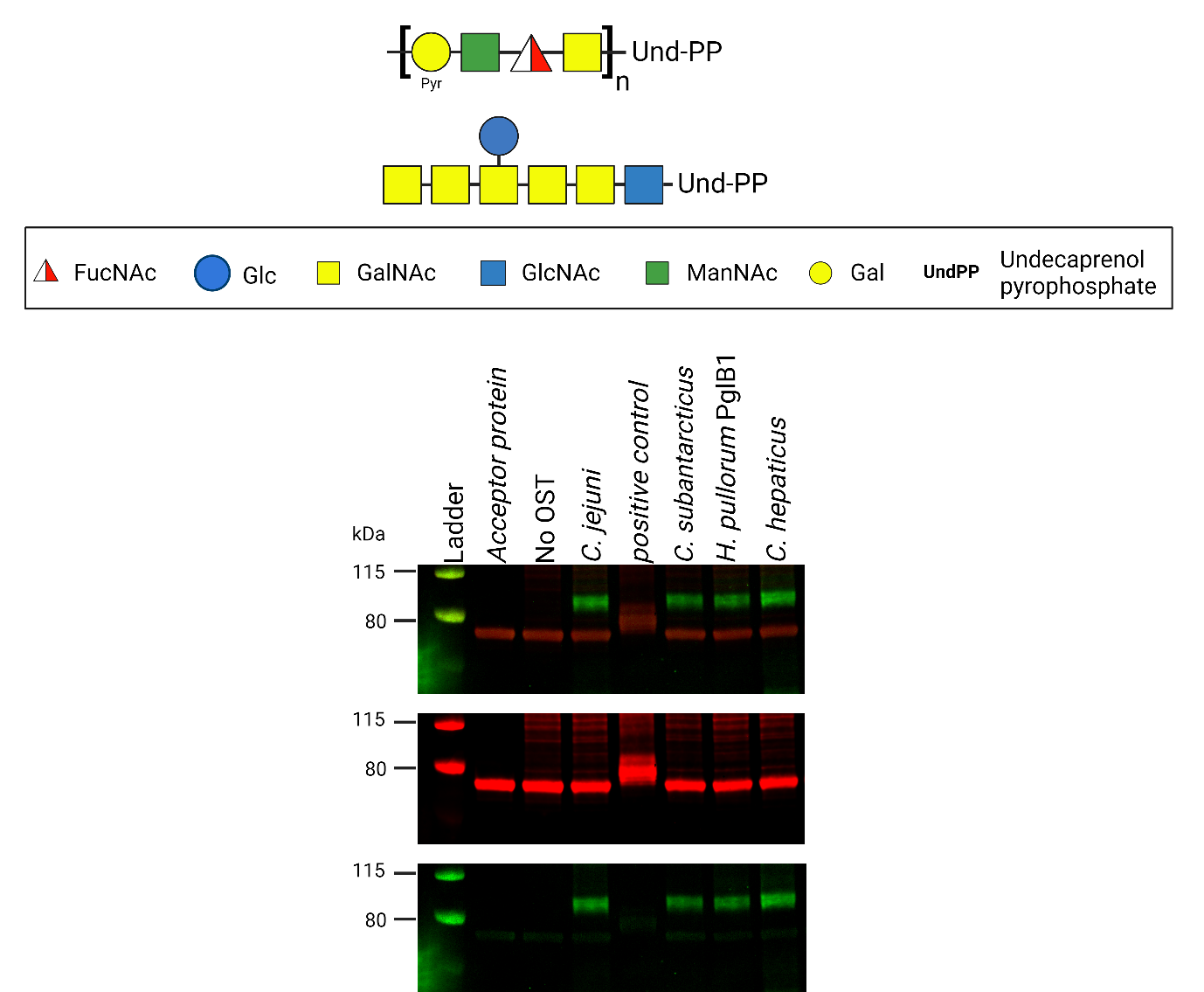


**Figure** **S11.** **Cell free glycosylation of *Streptococcus pneumonia serotype 4*.** expressed from *E. coli* W311O, with GalNAc and GlcNAc reducing end sugars. No OST consists of all substrates apart from OST. *C. jejuni* PglB was used as a positive control. Well labelled acceptor protein consists of acceptor protein only. Green and red bands represent glycan and protein respectively.


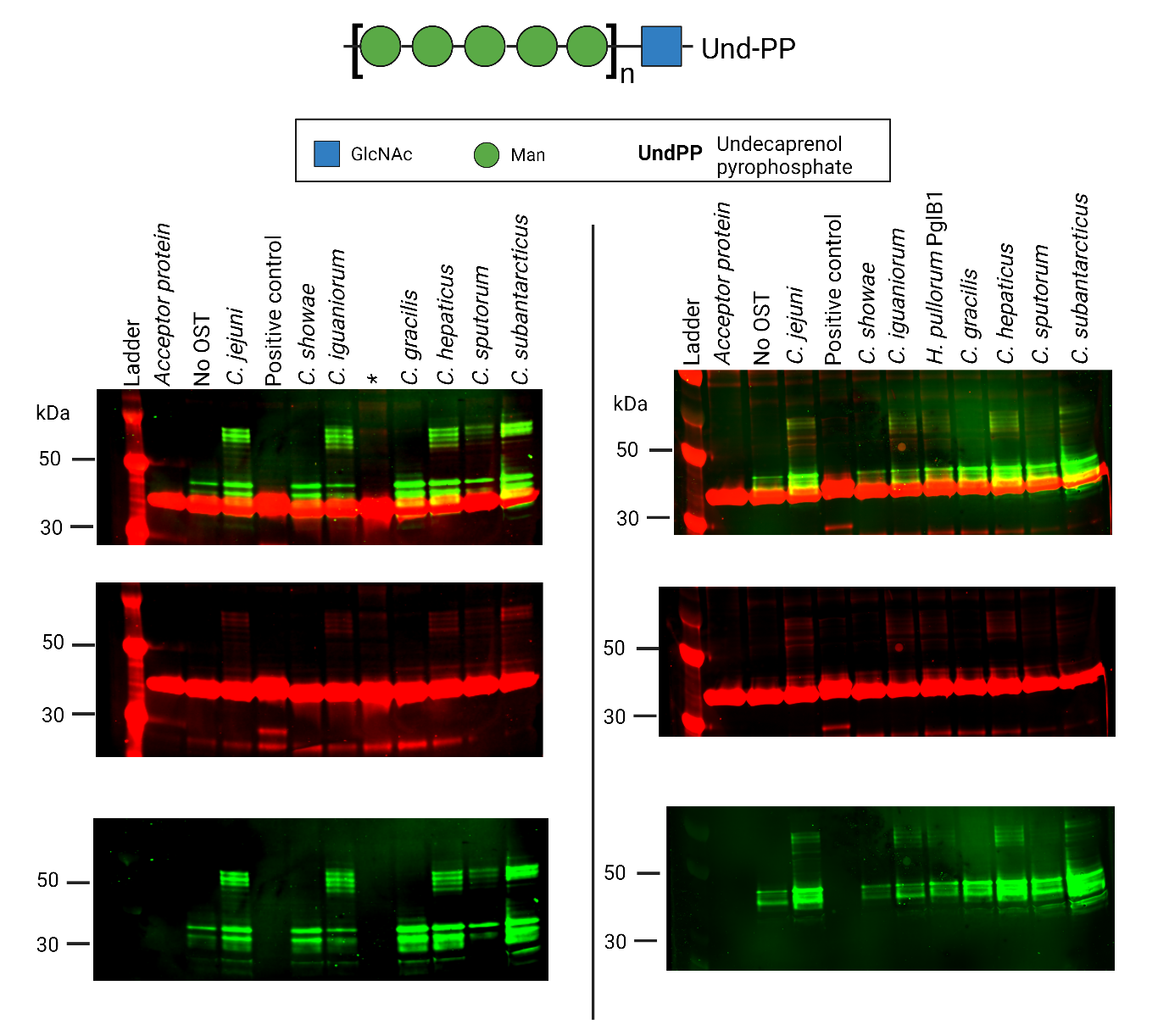


**Figure** **S12. Cell-free glycosylation of** ***E. coli* O9 antigen**. *E. coli* O9 antigen, expressed natively with GalNAc reducing end sugar. *C. jejuni* PglB with *C. jejuni* heptasaccharide expressed in *E. coli* CLM24 was used as a positive control. Acceptor protein consists of acceptor protein only, and no OST or glycan. Green and red bands represent glycan and protein respectively. * did not add *H. pullorum* PglB1 to the well.


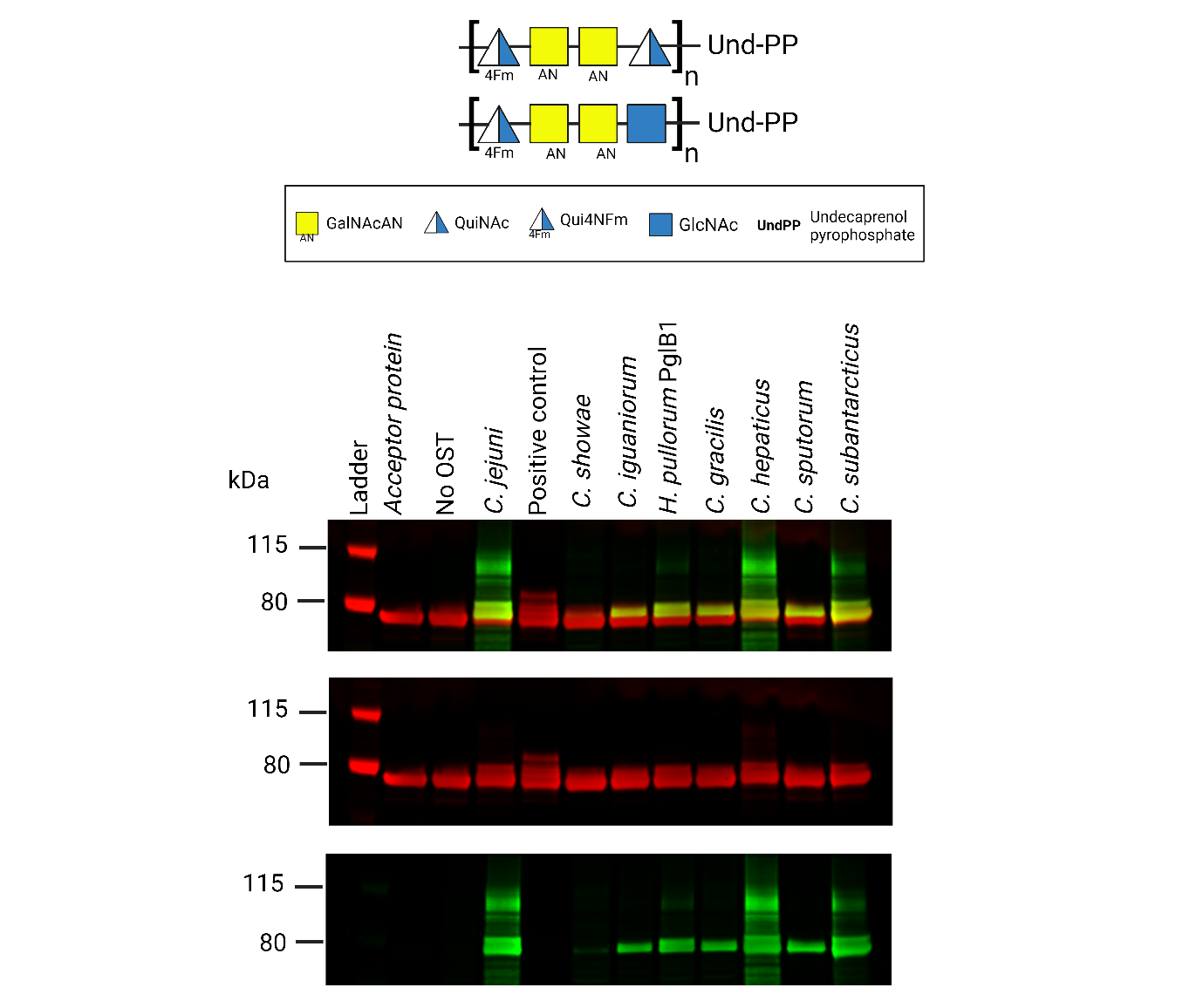


**Figure** **S13. Cell-free glycosylation of *E. coli* DH5alpha (wecA and waal +ve) with GlcNAc and Qui4NFm reducing end sugars.** *C. jejuni* PglB with *C. jejuni* heptasaccharide expressed in *E. coli* CLM24 was used as a positive control. Acceptor protein consists of acceptor protein only, and no OST or glycan. Green and red bands represent glycan and protein respectively.

**Table S7.** Densitometry measurements, along with normalisation analysis of cell-free glycosylation data of *Streptococcus pneumonia serotype 4* expressed from *E. coli* W3110, with GalNAc and GlcNAc reducing end sugars.

| OST | replicate 1 | replicate 2 | Normalised replicate 1 | | Normalised replicate 2 | | p value anova t test | | |
| --- | --- | --- | --- | --- | --- | --- | --- | --- | --- |
|  | Glycan raw densitometry (Area) | Glycan raw densitometry (Area) | Fold change | % difference to *C. jejuni* PglB | Fold change | % difference to *C. jejuni PglB* | Average % difference to *C. jejuni* PglB | SD of percent value | p value |
| *C. jejuni* | 61846.9 | 29528.3 | 0.0 | 0.0 | 0.0 | 0.0 | 0.0 | 0.0 |  |
| *H. pullorum PglB1* | 53177.2 | 27785.2 | 0.9 | 86.0 | 0.9 | 94.1 | 90.0 | 5.7 | 0.1 |
| *C. hepaticus* | 62620.9 | 27778.8 | 1.0 | 101.3 | 0.9 | 94.1 | 97.7 | 5.1 | 0.6 |
| *C. subantarcticus* | 52295.3 | 27779.5 | 0.8 | 84.6 | 0.9 | 94.1 | 89.3 | 6.7 | 0.2 |

**Table S8.** Densitometry measurements, along with normalisation analysis of *E. coli* O9 antigen, expressed natively with GalNAc reducing end sugar.

| OST | replicate 1 | replicate 2 | replicate 3 | Normalised replicate 1 | | Normalised replicate 2 | | Normalised replicate 3 | | p value anova t test | | |
| --- | --- | --- | --- | --- | --- | --- | --- | --- | --- | --- | --- | --- |
|  | Glycan raw densitometry (Area) | Glycan raw densitometry (Area) | Glycan raw densitometry (Area) | Fold change | % difference to *C. jejuni* PglB | Fold change | % difference to *C. jejuni PglB* | Fold change | % difference to *C. jejuni PglB* | Average % difference to *C. jejuni* PglB | SD of perecet value | p value |
| *C. jejuni* | 22682.6 | 8137.1 | 12205.7 | 0.0 | 0.0 | 0.0 | 0.0 | 0.0 | 0.0 | 0.0 |  |  |
| *C. iguaniourm* | 22731.3 | 9679.7 | 16423.3 | 1.0 | 100.2 | 1.2 | 119.0 | 1.3 | 134.6 | 117.9 | 17.2 | 0.1 |
| *C. hepaticus* | 19902.9 | 23607.3 | 13777.9 | 0.9 | 87.7 | 2.9 | 290.1 | 1.1 | 112.9 | 163.6 | 110.3 | 0.4 |
| *C. subantarcticus* | 19562.9 | 8201.8 | 14009.6 | 0.9 | 86.2 | 1.0 | 100.8 | 1.1 | 114.8 | 100.6 | 14.3 | 0.9 |

**Table S9.** Densitometry measurements, along with normalisation analysis of *Francisella* O antigen expressed in *E. coli* DH5alpha (wecA and Waal +ve).

| OST | replicate 1 | replicate 2 | Normalised replicate 1 | | Normalised replicate 2 | | p value anova t test | | |
| --- | --- | --- | --- | --- | --- | --- | --- | --- | --- |
|  | Glycan raw densitometry (Area) | Glycan raw densitometry (Area) | Fold change | % difference to *C. jejuni* PglB | Fold change | % difference to *C. jejuni PglB* | Average % difference to *C. jejuni* PglB | SD of perecet value | p value |
| *C. jejuni* | 53599.2 | 14459.9 | 0.0 | 0.0 | 0.0 | 0.0 | 0.0 | 0.0 |  |
| *C. showae* | 4421.2 | 675.4 | 0.1 | 8.2 | 0.0 | 4.7 | 6.5 | 2.5 | <0.0001 |
| *C. iguaniourm* | 11710.2 | 5012.7 | 0.2 | 21.8 | 0.3 | 34.7 | 28.3 | 9.1 | 0.0 |
| *H. pullorum PglB1* | 28166.7 | 7464.2 | 0.5 | 52.6 | 0.5 | 51.6 | 52.1 | 0.7 | <0.0001 |
| *C. gracillis* | 25883.4 | 5686.6 | 0.5 | 48.3 | 0.4 | 39.3 | 43.8 | 6.3 | 0.0 |
| *C. hepaticus* | 56339.9 | 11725.4 | 1.1 | 105.1 | 0.8 | 81.1 | 93.1 | 17.0 | 0.6 |
| *C. sputorum* | 29012.9 | 7063.0 | 0.5 | 54.1 | 0.5 | 48.8 | 51.5 | 3.7 | 0.0 |
| *C. subantarcticus* | 53776.0 | 12845.9 | 1.0 | 100.3 | 0.9 | 88.8 | 94.6 | 8.1 | 0.4 |

**Table S10.** Densitometry measurements of Cell-free glycosylation data for *C. jejuni* heptasaccharide expressed from *E. coli* CLM24, with DiNAcBac and GlcNAc reducing end sugars

| OST | Glycan raw densitometry (Area) |
| --- | --- |
| *C. jejuni* | 103611.2 |
| *C. showae* | 11396.7 |
| *C. iguaniourm* | 64168.5 |
| *H. pullorum PglB1* | 53610.1 |
| *C. gracillis* | 74139.2 |
| *C. hepaticus* | 100859.9 |
| *C. sputorum* | 75315.6 |
| *C. subantarcticus* | 93595.6 |

**Table** **S11.** Densitometry measurements of Cell-free glycosylation data for *C. jejuni* heptasaccharide expressed from SDB1, with a DiNAcBac reducing end sugar.

| OST | Glycan raw densitometry (Area) |
| --- | --- |
| *C. jejuni* | 41373.9 |
| *C. showae* | 16699.4 |
| *C. iguaniourm* | 45393.1 |
| *H. pullorum PglB1* | 17797.3 |
| *C. gracillis* | 57773.6 |
| *C. hepaticus* | 54828.5 |
| *C. sputorum* | 70677.9 |
| *C. subantarcticus* | 46186.8 |

**Table** **S12.** Densitometry measurements, along with normalisation analysis of Cell-free glycosylation of *C. jejuni* heptasaccharide expressed from *E. coli* SDB1.

|  | OST | Glycan raw densitometry (Area) | Average | SD | p value |
| --- | --- | --- | --- | --- | --- |
| replicate 1 | *C. jejuni* | 41033.7 | 42632.4 | 2810.9 | 0.004 |
| replicate 2 | *C. jejuni* | 45878.0 |  |  |  |
| replicate 3 | *C. jejuni* | 40985.5 |  |  |  |
| replicate 1 | *C. hepaticus* | 55749.6 | 53926.1 | 1851.5 |  |
| replicate 2 | *C. hepaticus* | 53980.7 |  |  |  |
| replicate 3 | *C. hepaticus* | 52047.8 |  |  |  |

**Table** **S13.** Densitometry measurements, along with normalisation analysis of Cell-free glycosylation of *C. jejuni* heptasaccharide expressed from *E. coli* CLM24.

|  | OST | Glycan raw densitometry (Area) | Average | SD | p value |
| --- | --- | --- | --- | --- | --- |
| replicate 1 | *C. jejuni* | 49517.1 | 53086.0 | 3421.9 | 0.131 |
| replicate 2 | *C. jejuni* | 53402.1 |  |  |  |
| replicate 3 | *C. jejuni* | 56338.9 |  |  |  |
| replicate 1 | *C. hepaticus* | 78774.3 | 66953.7 | 12187.4 |  |
| replicate 2 | *C. hepaticus* | 67656.7 |  |  |  |
| replicate 3 | *C. hepaticus* | 54430.0 |  |  |  |

**Table S14.** Densitometry measurements, along with normalisation analysis of *C. jejuni* and *C. hepaticus* PglB transfer ability of *C. jejuni* heptasaccharide to EPA in live *E. coli* SDB1 in LB broth.

|  | OST | Glycan raw densitometry (Area) | Average | SD | p value |
| --- | --- | --- | --- | --- | --- |
| Replicate 1 | *C. hepaticus* | 4255.4 | 8740.9 | 6154.8 | 0.01 |
| Replicate 2 | *C. hepaticus* | 6209.7 |  |  |  |
| Replicate 3 | *C. hepaticus* | 15757.7 |  |  |  |
| Replicate 1 | *C. jejuni* | 58069.7 | 68322.6 | 21833.9 |  |
| Replicate 2 | *C. jejuni* | 53502.2 |  |  |  |
| Replicate 3 | *C. jejuni* | 93396.0 |  |  |  |

**Table S15.** Densitometry measurements, along with normalisation analysis of *C. jejuni* and *C. hepaticus* PglB transfer ability of *C. jejuni* heptasaccharide to EPA in live *E. coli* SDB1 in 2YPTG broth.

|  | OST | Glycan raw densitometry (Area) | Average | SD | p value |
| --- | --- | --- | --- | --- | --- |
| Replicate 1 | *C. jejuni* | 27286.1 | 21627.4 | 4916.0 | 0.266 |
| Replicate 2 | *C. jejuni* | 19188.5 |  |  |  |
| Replicate 3 | *C. jejuni* | 18407.7 |  |  |  |
| Replicate 1 | *C. hepaticus* | 31319.4 | 28028.0 | 7037.3 |  |
| Replicate 2 | *C. hepaticus* | 32816.6 |  |  |  |
| Replicate 3 | *C. hepaticus* | 19948.1 |  |  |  |
